# Alternative splicing in the DBD linker region of p63 modulates binding to DNA and iASPP in vitro

**DOI:** 10.1038/s41419-024-07320-2

**Published:** 2025-01-06

**Authors:** Rebecca Lotz, Christian Osterburg, Apirat Chaikuad, Sabrina Weber, Masato Akutsu, Anne Christin Machel, Ulrike Beyer, Jakob Gebel, Frank Löhr, Stefan Knapp, Matthias Dobbelstein, Xin Lu, Volker Dötsch

**Affiliations:** 1 Institute of Biophysical Chemistry and Center for Biomolecular Magnetic Resonance, Goethe University, 60438 Frankfurt, Germany; 2 Institute of Pharmaceutical Chemistry, Goethe University, 60438 Frankfurt, Germany; 3 Structural Genomics Consortium (SGC), Goethe University, 60438 Frankfurt, Germany; 4 Institute of Molecular Oncology, Center of Molecular Biosciences, University of Göttingen, Göttingen, Germany; 5 Buchmann Institute for Molecular Life Sciences, Goethe University, 60438 Frankfurt, Germany; 6 Research Center for Advanced Analysis, National Agriculture and Food Research Organization (NARO), Tsukuba, Ibaraki Japan; 7 Ludwig Institute for Cancer Research, Nuffield Department of Clinical Medicine, University of Oxford, Oxford, OX3 7DQ UK

**Keywords:** Structural biology, Biophysical chemistry

## Abstract

The transcription factor p63 is expressed in many different isoforms as a result of differential promoter use and splicing. Some of these isoforms have very specific physiological functions in the development and maintenance of epithelial tissues and surveillance of genetic integrity in oocytes. The ASPP family of proteins is involved in modulating the transcriptional activity of the p53 protein family members, including p63. In particular, iASPP plays an important role in the development and differentiation of epithelial tissues. Here we characterize the interaction of iASPP with p63 and show that it binds to the linker region between the DNA binding domain and the oligomerization domain. We further demonstrate that this binding site is removed in a splice variant of p63 where a stretch of five amino acids is replaced with a single alanine residue. This stretch contains a degenerate class II SH3 domain binding motif that is responsible for interaction with iASPP, as well as two positively charged amino acids. Moreover, the concomitant loss of the charged amino acids in the alternatively spliced version decreases the affinity of p63 to its cognate DNA element two- to threefold. mRNAs encoding full-length p63, as well as its alternatively spliced version, are present in all tissues that we investigated, albeit in differing ratios. We speculate that, through the formation of hetero-complexes of both isoforms, the affinity to DNA, as well as the interaction with iASPP, can be fine-tuned in a tissue-specific manner.

## Introduction

The ASPP family consists of the three members ASPP1, ASPP2 and iASPP, which are mostly known for modulating the function of p53, thereby directing cell fate decision [1–3]. ASPP1 and ASPP2 enhance the pro‐apoptotic activity of p53 [2], whereas iASPP inhibits it [3]. Despite their antagonizing functions, the ASPP family members share a highly homologous C‐terminal domain (CTD) which mediates the direct interaction with p53 and consists of four ankyrin repeats (ARs) conjoined with an SH3 domain [4]. Generally, the CTDs interact with the DNA binding domains (DBDs) of the p53 family members, but the ASPP2 and iASPP CTD also bind the unfolded linker region of p53 between the DBD and the oligomerization domain (OD) [5, 6]. A third, iASPP-exclusive interaction site that contains several SH3 domain binding motifs (PxxP) is located in the proline-rich domain (PRD) of p53 [7].

**Fig. 1:**
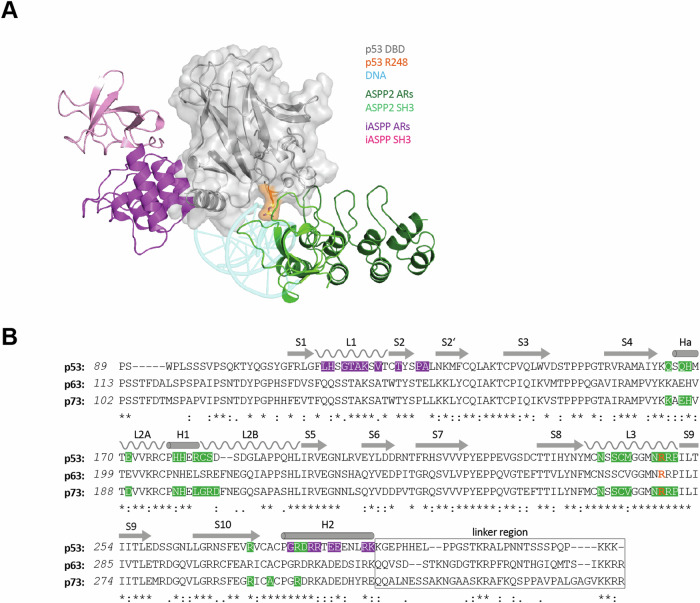
Binding sites of ASPP family members and the DBDs of p53 family members. **A** Superimposed structures of the p53 DBD (grey) bound to DNA (light blue), ASPP2 CTD (green) and iASPP CTD (purple). The SH3 domain and the ankyrin repeats of the CTDs are shown in light and dark colour, respectively. The residue R248 of p53 (orange) directly contacts DNA and the SH3 domain of ASPP2. Zinc is displayed as a grey sphere (PDB: 2AC0, 1YCS and 6RZ3). **B** Sequence alignment of the p53 family DBDs and the proximate linker region. The secondary structure elements of the DBDs are shown and labelled accordingly. Residues involved in binding ASPP2 CTD and iASPP CTD are shaded in green and purple, respectively. The arginine (p53: R248, p63: R279, p73: R268) involved in the interaction with DNA and ASPP2 is highlighted in orange. The linker region (black box) of p53 is known to be bound by iASPP CTD.

Despite the high sequence and structural homology, the CTDs bind different interfaces of the DBD. The ASPP1 and ASPP2 CTDs interact with the DNA binding interface of the p53 DBD with affinities in the lower µM range, which involves several residues of the p53 DBD, that are frequently mutated in cancer e.g. R248 (Fig. 1A) [2, 4]. Consequently, the R248W DNA contact hotspot mutation does not only impair DNA binding but also the interaction with ASPP2. Binding of ASPP2 thus blocks contact with DNA (mechanistically it is not understood how this binding stimulates p53 activity [2]). In contrast, the iASPP CTD binds to the loop-sheet-helix region of the p53 DBD without targeting the residues directly interacting with the DNA [8]. The only effect on DNA binding is the displacement of the L1 loop (Fig. 1A) which positions the side chain of K120 to contact the second base of the PuPuPuC(A/T) (quarter site) DNA binding site. This displacement modifies the sequence preference of p53 and changes the transcriptional program, suppressing transcription of genes involved in apoptosis such as *Fas*, *TIGAR* or *PMAIP1*. Of note, depletion of iASPP results in a strong p53-dependent induction of TIGAR, even in the absence of any (stress) stimuli suggesting that iASPP also contributes to the regulation of p53 activity under non-stressed conditions [8].

The DNA binding domain of p53 shows a high sequence identity with the other two members of the p53 protein family, p63 and p73 (Fig. 1B) [9]. The DNA binding interface, in particular, is highly conserved. Not surprisingly, ASPP1 and ASPP2 have been shown to bind to p63 and p73 as well [5, 10]. In contrast, the L1 loop region is less conserved, with key residues crucial for iASPP binding to the p53 DBD, such as H115, being only present in p53 [8]. This suggests that the mode of interaction between the p53 DBD and iASPP is unique. Nonetheless, the binding of iASPP to the DBDs of p63 and p73 has also been reported [10].

Mutational data and functional investigations have further demonstrated that iASPP is involved in regulating the activity of p63 as well. However, neither the exact interaction interface(s) between p63 and iASPP nor the regulatory mechanisms are understood. p63 is highly expressed in the basal compartment of stratified epithelial tissues [11] and its inactivation results in severe developmental defects, including limb truncations and the lack of a multi-layered skin, as well as of other epithelial structures [12, 13]. Interestingly, iASPP co-localizes with p63 in the nucleus of human basal epithelial cells and is involved in the regulation of p63 and AP1 target genes important for the maintenance of tissue homeostasis [14].

Here we demonstrate that the main binding site of iASPP on p63 is in the linker region between the DBD and the OD and that this region is lost due to splicing in a so far understudied isoform of p63.

## Results

**Fig. 2:**
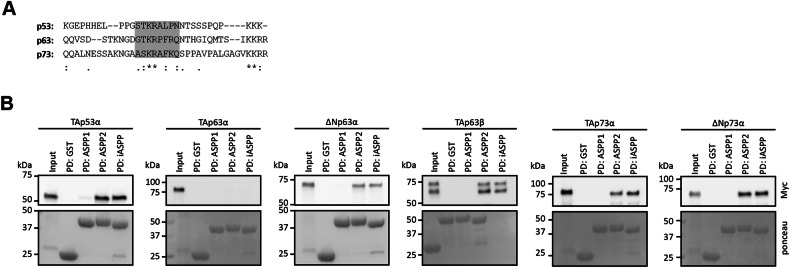
Interaction studies between p53 family members and ASPP family members. **A** Sequence alignment of the p53 family linker region. The core region of the linker (based on sequence homology to the p53 linker region) is shaded in grey. **B** Pull-down assay of p53 family member isoforms with GST-tagged ASPP family CTDs and GST alone as bait. N terminally Myc-tagged proteins were in-vitro translated using RRL. Input and pull-down (PD) samples were analysed by WB using an α-Myc antibody. The bait protein in the PD samples was detected by ponceau staining of the WB membrane.

The iASPP CTD was reported to preferentially bind the linker region between the DBD and OD of p53 [6]. An alignment revealed a short sequence within this region that is conserved to some degree among all p53 family members (Fig. 2A). As a first interaction study we performed pulldown assays with recombinantly expressed and purified ASPP family CTDs fused to a GST‐tag as bait and in vitro translated p53 family members. p63 and p73 exist in several different isoforms that result from the combination of different promotors, creating different N-termini and splicing events at the C-terminus [11, 15]. As these isoforms have quite different properties we used a representative set including p53 and major p63 and p73 isoforms for the pulldown experiments. All proteins except TAp63α were bound by ASPP2 and iASPP (Fig. 2B, Supplementary Fig. S1). In contrast to all other p53 family members that form tetramers via their OD, TAp63α adopts a closed dimeric state [16]. The absence of an interaction between TAp63α and ASPP2 confirms earlier results suggesting that the interface bound by ASPP2 is occluded in the closed dimeric conformation [17]. The pulldown experiments, however, also revealed that iASPP cannot bind to TAp63α, suggesting that the linker region is not accessible in the closed state as well. Surprisingly, no interaction of any p53 family member with ASPP1 could be verified even though the affinity of its CTD for the p53 family DBDs was reported to be in the sub µM range [5].

### The ΔExon8 variant has a reduced binding affinity to DNA

**Fig. 3:**
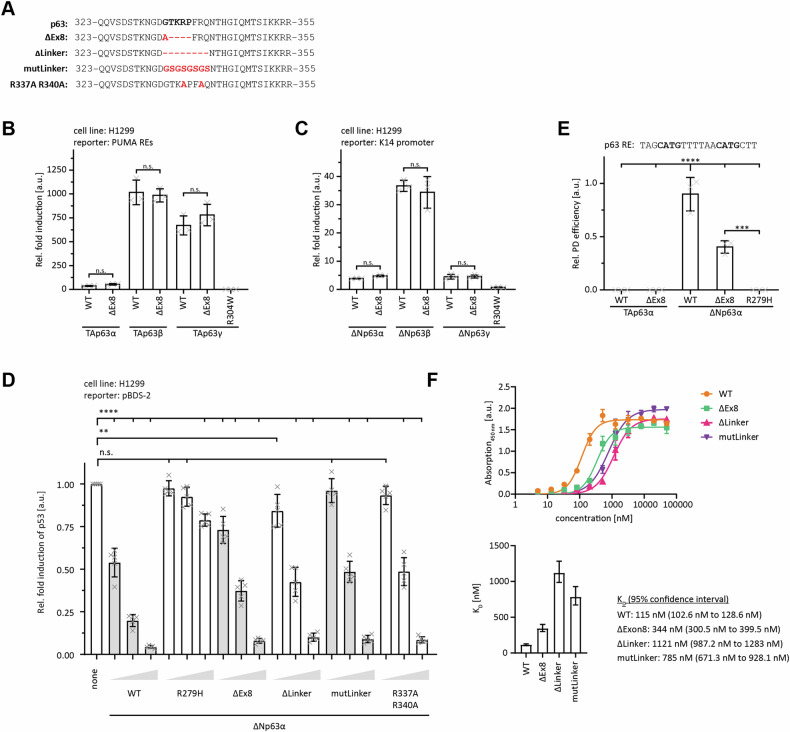
The linker region modulates the interaction with DNA. **A** Alignment of the canonical and ΔExon8 linker region of p63 with the three indicated variants used for the following assays. **B** Luciferase reporter assay of TAp63 isoforms with the WT or ΔExon8 (ΔEx8) linker region on the PUMA response element. The R304W DNA contact mutant served as a negative control. H1299 cells were transiently transfected with the respective luciferase reporter plasmids and the N-terminally Myc-tagged proteins. **C** Luciferase reporter assay of ΔNp63 isoforms with the WT or ΔExon8 (ΔEx8) linker region on the K14 promoter. The R304W DNA contact mutant served as a negative control. H1299 cells were transiently transfected with the respective luciferase reporter plasmids and the N-terminally Myc-tagged proteins. **D** Luciferase reporter assay of ∆Np63α WT and indicated variants displacing p53 from a pBDS-2 reporter. Luciferase reporter plasmid and Myc-tagged p53 were transiently transfected into H1299 cells along with increasing amounts of the indicated ∆Np63α variants. **E** DNA pull-down assay of TAp63α and ΔNp63α, carrying either the WT or ΔExon8 (ΔEx8) linker region, with the 20 bp p63 RE of as bait. The R279H DNA contact mutant served as a negative control. N-terminally Myc-tagged proteins were in-vitro translated using RRL. Input (IP) and pull-down (PD) samples were analysed by WB using an α-Myc antibody. For the relative pull-down efficiency each PD signal was normalized to the IP signal and p63 WT was set to 1. **F** Elisa assay of purified p63 DBD-TD WT (AA 123–416) and the indicated variants binding to an immobilized 20 bp RE of the p21 promotor. Measurements were performed three times and binding curves were fitted with a non-linear, least square regression using a single-exponential one-site binding model with Hill slope (upper). Determined DNA binding affinities were plotted in a bar diagram with error bars corresponding to the 95% confidence interval (lower). **(B-E)** The bar diagrams show the mean values and error bars the corresponding SD (*n* = 3). Statistical significance was assessed by ordinary one-way ANOVA (n.s.*P* > 0.05, *P ≤ 0.05, **P ≤ 0.01, ***P ≤ 0.001, ****P ≤ 0.0001).

One so far understudied isoform of p63 is created by splicing events in the linker region between the DBD and the OD. This isoform was initially found by the McKeon lab [11] and later cloned again from a human placenta cDNA library [18]. In this isoform (called ΔExon8, with exon 8 referring to the TAp63α isoform) a short stretch of five amino acids (GTKRP), located C-terminally to the DBD is exchanged for a single alanine residue (Fig. 3A). Interestingly, the exchanged five residues are part of the conserved sequence in the linker region, predicted to interact with iASPP. To investigate the importance of this sequence we first analysed the general effect of this splice variation on the properties of different p63 isoforms. The ΔExon8 splice variation did not affect the oligomeric state of TAp63α or ΔNp63α (Supplementary Fig. S2A), nor the fold or stability of the isolated DBD (Supplementary Fig. S2B, C). The transcriptional activity of the ΔExon8 isoform was unaltered in transiently transfected H1299 cells, on both the PUMA promotor (TAp63 isoforms) and on the K14 promotor (ΔNp63 isoforms) (Fig. 3B, C; Supplementary Fig. S2D, E). However, transactivation assays based on transient overexpression of transcription factors are sensitive to saturation effects. In a recent study of the transcriptional activity of p63 mutants identified in ELA syndrome patients, we originally did not see a difference between several of them and the wild-type protein. For further investigations, we had designed a competition assay in which the transcriptional activity of p53 was monitored in the presence of increasing amounts of different ΔNp63α variants, which binds to the same DNA binding sites but has only a minimal transcriptional activity. DNA binding of the mutants can thus be monitored by their ability to compete with p53 [19]. We adapted this assay to investigate the effect of the ΔExon8 splice variation. For a negative control, we also included an R279H DNA contact mutant, which corresponds to R248W in p53 and causes the ELA developmental syndrome in humans [19, 20]. As can be seen in Fig. 3D (and Supplementary Fig. S2F), low concentrations of the ΔExon8 splice variant are less efficient in competing with p53 than p63 WT. Deleting the core linker region (ΔLinker, including F339-Q341 which is the sequence with the highest sequence homology to the p53 linker region) or replacing this region with the same length of a glycine-serine stretch (mutLinker) surprisingly had the same effect. This result suggests that not the reduced linker length is responsible for the decrease in DNA binding capacity of the ΔExon8 splice variation but the exact amino acid sequence. This linker sequence contains two Arg residues whose positive charge can potentially contribute to DNA binding. Mutating both to Ala (R337A R340A) indeed reduced the ability of the mutated ΔNp63α to compete with p53, resembling the ΔExon8 splice variation (note, that only R337 is removed by the splicing event; R340 is part of the extended linker sequence with homology to the p53 linker region).

To further investigate the interaction with the DNA directly, we performed pulldown experiments of ΔNp63α and ΔNp63α ΔExon8 with a p63 DNA response element. These results indeed showed a significant reduction of the DNA binding affinity (Fig. 3E; Supplementary Fig. S2G).

To obtain more quantitative data we also measured the DNA binding affinity using an ELISA assay. The p63 construct containing the DBD and the OD domains, including the linker region bound a p21 promoter containing oligonucleotide with a K_D_ of 115 nM. The ΔExon8 version of this construct as well as the deletion of the core linker sequence or its replacement with a gycine-serine stretch reduced the affinity three to tenfold (Fig. 3F). These results again suggest that the specific amino acids of the linker region contribute to DNA binding.

### The ΔExon8 variant loses the ability to interact with iASPP

**Fig. 4:**
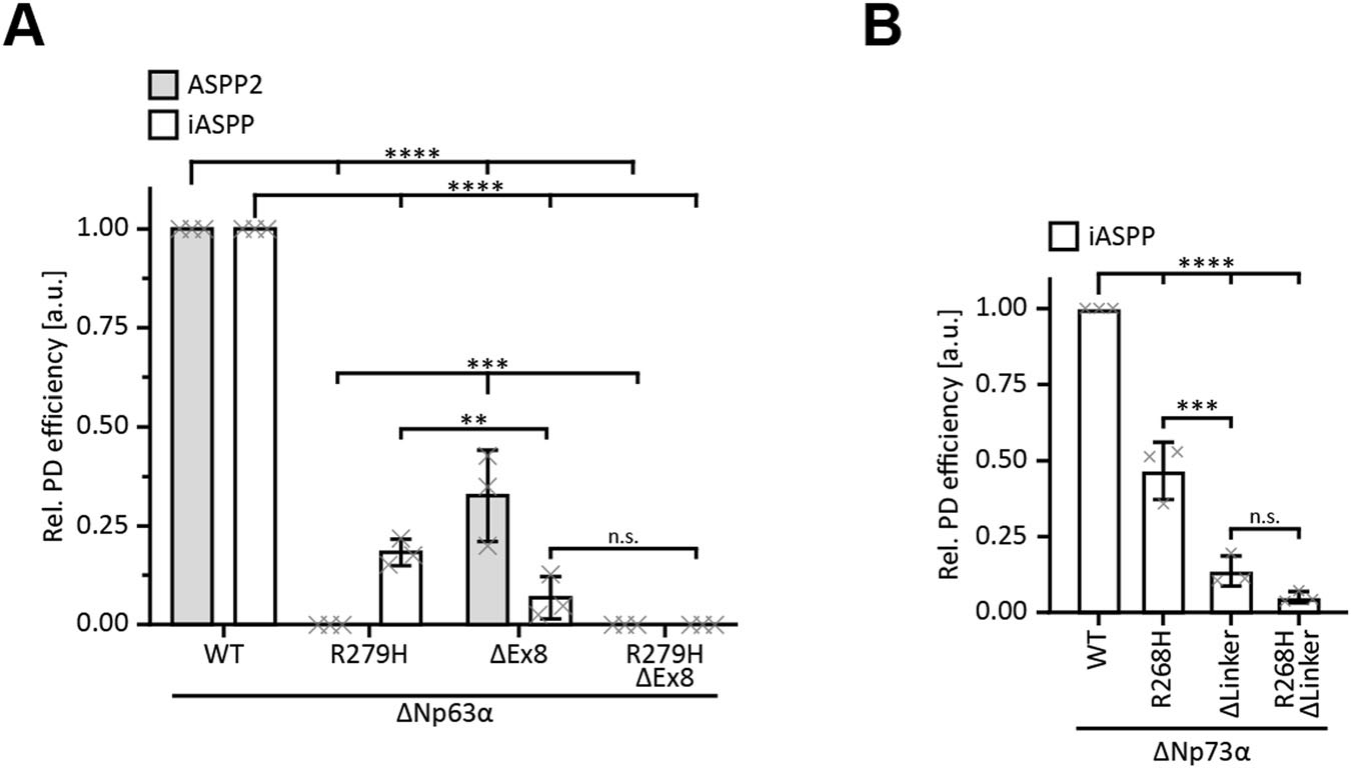
The linker region modulates the interaction with members of the ASPP family. **A** Pull-down assay of ΔNp63α WT and indicated variants with GST-tagged ASPP2 (grey) and iASPP CTDs (white) as bait. N-terminally Myc-tagged proteins were in-vitro translated using RRL. Input and pull-down (PD) samples were analysed by WB using an α-Myc antibody. For the relative pull-down efficiency, the PD signals were normalized to the IP signals and p63 WT was set to 1 for ASPP2 and iASPP individually. **B** Pull-down assay of ΔNp73α WT and indicated variants with GST-tagged iASPP CTD as bait. N-terminally Myc-tagged proteins were in-vitro translated using RRL. Input and pull-down (PD) samples were analysed by WB using an α-Myc antibody. For the relative pull-down efficiency, the PD signals were normalized to the IP signals and p73 WT was set to 1. **A, B** The bar diagrams show the mean relative pull-down efficiency and error bars the corresponding SD (*n* = 3). Statistical significance was assessed by ordinary one-way ANOVA (n.s.: *P* > 0.05, *: *P* ≤ 0.05, **: *P* ≤ 0.01, ***: *P* ≤ 0.001, ****: *P* ≤ 0.0001).

To experimentally test if the ΔExon8 splice variation is important for the interaction with iASPP as well, we performed pulldown assays as described above. The introduction of R279H in ΔNp63α completely abolished the interaction with ASPP2, while only partially affecting the binding to iASPP (Fig. 4A; Supplementary Fig. S3A). The deletion in the linker region in the ΔExon8 isoform virtually impaired binding to iASPP but impacted the interaction with ASPP2 to a lesser extent. Accordingly, the double modified version ΔNp63α R279H ΔExon8 neither bound ASPP2 nor iASPP. These results suggest not only that the CTDs of ASPP2 and iASPP interact with both the DBD and the linker region of p63, but also that, as reported for p53, ASPP2 preferentially binds the DBD and iASPP the linker region. The preference of the ASPP2 CTD for the p63 DBD was expected due to the high conservation of the interface between p53 family members. In contrast, for the iASPP CTD, the DNA binding interface seems to provide an alternative interaction site since the region around the loop L1, reported to be the interaction site with the p53 DBD, is not conserved in p63. For iASPP, very similar results were also obtained in pulldown experiments with ΔNp73α (Fig. 4B; Supplementary Fig. S3B). Mutations in the DNA binding interface (R268H) partially impaired the binding of iASPP. A stronger effect was seen for deleting the corresponding linker region and the double mutation impaired binding completely.

**Fig. 5:**
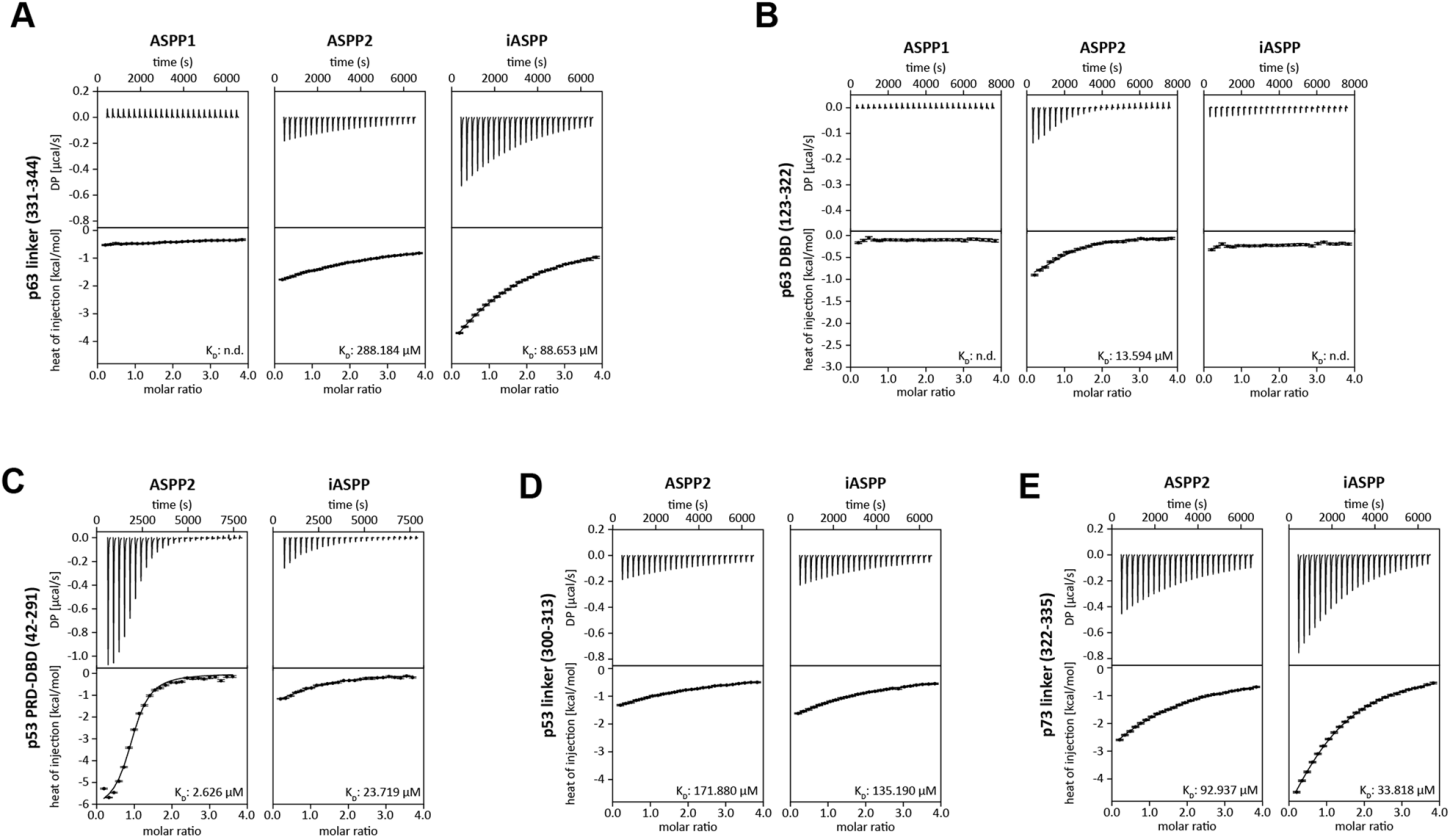
ASPP2 and iASPP prefer different binding sites. **A** ITC results of the p63 linker peptide titrated to ASPP family CTDs. **B** ITC results of the p63 DBD titrated to ASPP family CTDs. **C** ITC results of the p53 DBD titrated to ASPP2 and iASPP CTDs. **D** ITC results of the p53 linker peptide titrated to ASPP2 and iASPP CTDs. **E** ITC results of the p73 linker peptide titrated to ASPP2 and iASPP CTDs. **A**–**E** In each of the titration experiments the raw titration profiles are displayed in the top diagrams and integrated heat in the bottom diagram. Best fit of single-site binding model is shown as a solid black line with the resulting K_D_.

We further characterized the interaction of the ASPP family CTDs with p63 using isothermal titration calorimetry (ITC). These measurements confirmed quantitatively that ASPP2 binds with higher affinity to the DBD compared to the linker peptide while the reverse was found for iASPP without a detectable interaction between the p63 DBD and iASPP CTD (although the pulldown experiments in Fig. 4A suggested that an interaction with the DBD exists) (Fig. 5A, B). No interaction was seen with ASPP1, also confirming the pulldown results. For comparison, we measured the affinity to the p53 PRD-DBD fusion construct and the linker region (Fig. 5C, D). ASPP2 binds tightly to the p53 PRD-DBD while the affinity for iASPP is ten times lower. Both bind only weakly to the linker region, with affinities similar to those for the p63 linker. The strongest interaction for the linker peptide for both ASPP2 and iASPP CTDs, however, was found for p73 (Fig. 5E).

Combined, the pulldown and the ITC data show that the main interaction site for the CTD of iASPP with p63 is the linker region that is removed in the ΔExon8 isoform. The interaction motif in the p63 linker region comprises a threonine and lysine residue, possible sites for posttranslational modifications. ITC measurements showed, however, that neither the phosphorylation of T335 nor the acetylation of K336 affect binding to the iASPP CTD (in contrast to the iASPP interaction with the p53 linker) (Supplementary Fig. S4).

### Structural characterization of the p63 – iASPP interaction

**Fig. 6:**
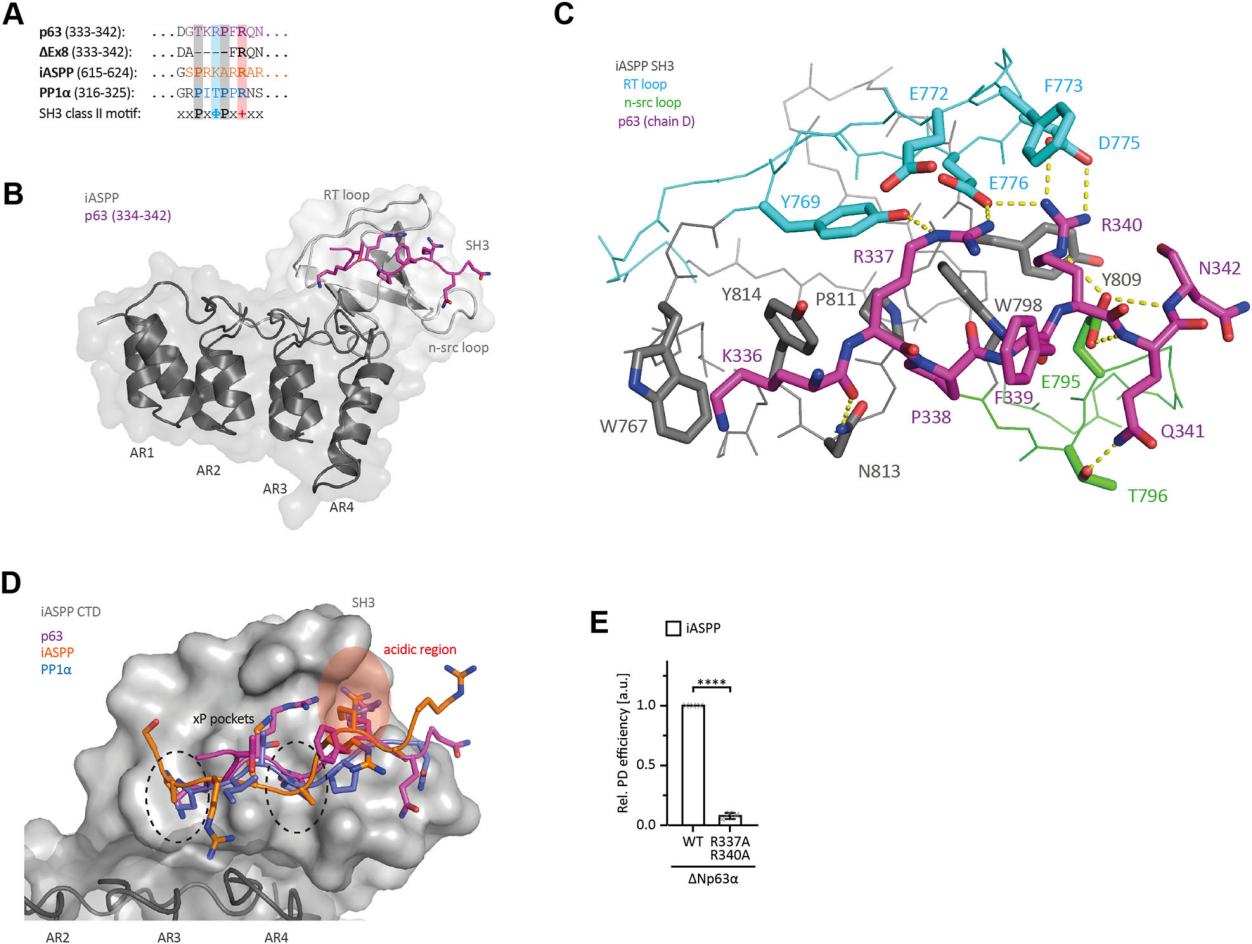
Structural characterization of the interaction of the linker region with iASPP. **A** Alignment of p63 linker region (AA 333 to 342), iASPP (AA 615 to 624) and PP1α (AA 316–325) peptides binding to the iASPP SH3 domain. Essential residues of the SH3 class II motif present in the peptides are highlighted in bold and color code (Φ: any aliphatic residue; +: any positively charged residue). This motif is deleted in ΔExon8 (AA 333 to 342). **B** Solved crystal structure of p63 (332–342)-iASPP CTD (625–828) fusion protein (p63 chain D and iASPP chain B) reveals the binding of the linear p63 motif (purple stick) to the SH3 domain of iASPP (light grey) involving both the RT and n-src loop. The ankyrin repeats (ANK1 to ANK4, dark grey) do not contribute to the interaction. Electron density was only observed for residue 334 to 342 of p63. See Table S2 for data collection and refinement statistics. **C** Detailed view of the interaction network between the p63 peptide (purple sticks; chain D) and the iASPP SH3 domain (chain B). The overall structure of the SH3 domain is shown in lines with important side chains depicted as sticks (SH3 core in grey, RT loop in cyan and n-src loop in green). Potential ionic and H-bonds are displayed as yellow dotted lines. **D** Superimposition of p63 (purple; chain D), iASPP (orange; PDB: 2VGE) and PP1α (blue; PDB: 6DCX) peptides bound to iASPP CTD (SH3 domain in light grey, ankyrin repeats in dark grey). The two xP pockets (dashed circle) and the acidic region (red) binding the two conserved prolines and the positive charged residue of the canonical SH3 motif, respectively, are highlighted. Sequences of the visible peptides are shown in (D) according to the colour code of the letters. **E** Pull-down assay of ΔNp63α WT and the R337A/R340A linker double mutant with GST-tagged iASPP CTD as bait. N-terminally Myc-tagged proteins were in-vitro translated using RRL. Input and pull-down (PD) samples were analysed by WB using an α-Myc antibody. For the relative pull-down efficiency, the PD signals were normalized to the IP signals and p63 WT was set to 1. The bar diagram shows the mean relative pull-down efficiency and error bars the corresponding SD (*n* = 3). Statistical significance was assessed by ordinary one-way ANOVA (n.s.: *P* > 0.05, *: *P* ≤ 0.05, **: P ≤ 0.01, ***: *P* ≤ 0.001, ****: *P* ≤ 0.0001).

NMR titration experiments had shown that ASPP2 and iASPP bind the p53 linker region via the SH3 domains of their CTDs [6]. Generally, SH3 domains bind short proline‐rich motifs. +xφPxxP and PxφPx+ are the canonical class I and class II SH3 domain binding motifs, respectively, with + being a positively charged residue and φ an aliphatic one [21, 22]. The class I peptides adopt the plus (+) orientation and the class II peptides the minus (‐) orientation with reversed N‐ and C‐termini. Sequence analysis of the p63 linker region revealed a degenerate class II SH3 domain binding motif, which is lost in the ΔExon8 isoform. It shows similarity to a class II motif found N-terminally to the CTD in iASPP itself (aa 617‐622, PRKARR) that was shown to bind to the SH3 domain of another iASPP molecule in the (-) orientation with a K_D_ of 45 µM (Fig. 6A) [10]. Relative to this iASPP peptide, the p63 peptide contains a positively charged residue, but instead of the second proline, it lacks the first one. We wanted to investigate the structural details of the interaction and solved the crystal structure of the iASPP CTD in complex with the p63 linker peptide (Fig. 6B). Since the K_D_ for the interaction is with ~90 µM relatively weak, we fused the p63 peptide (amino acids 332‐342) directly to the iASPP CTD (amino acids 625‐828). In the crystal structure, the p63 peptide of one fusion protein bound to the SH3 domain of a neighboring protein of the asymmetric unit. The p63 peptides of all four chains present within the asymmetric unit of the crystal lattice adopted an almost identical conformation with differences only in the orientation of T335, Q341 and F339 (Supplementary Fig. S5A). No electron density was observed for G332 and D333 at all, whereas G334 was only resolved in one chain. In detail, the p63 peptide adopted the conformation of a class II SH3 domain binding motif (PxφPx + ) despite the lack of one proline (Fig. 6C, D). P338 occupied the second xP pocket, whereas R340 contacted D775 and E776 of the RT loop and E795 of the n‐src loop which make up the acidic region of the SH3 domain. p63 R337, at the position of the aliphatic residue in the canonical class II motif, interacted with the RT loop as well by reaching towards E776 of the acidic region. The aliphatic moiety of the K336 side chain partially occupied the first xP pocket. The proximity of the W767 side chain could also allow for a cation‐π interaction via the ε‐amino group of K336. The mutation of N813 and Y814 resulted in only a two‐fold reduction of the affinity (Supplementary Fig. S5B) and acetylation had virtually no effect (Supplementary Fig. S4B) suggesting that K336 and the first xP pocket only slightly contribute to the interaction.

The degenerate SH3 domain-binding motif of iASPP and p63 each lack one proline, in contrast to the high‐affinity class II SH3 domain-binding motif of the PP1α C‐terminus (Fig. 6A) that binds iASPP with a sub-µM K_D_ (Supplementary Fig. S5C) [23]. Nevertheless, they adopted the same canonical class II conformation in the crystal structures (Fig. 6D). Although partially compensated by the additional contacts of the lysine and arginine side chains, the lack of a proline at the respective position explains the weak µM affinities which also results in virtual no interaction with the classical SH3 of Src kinase (Supplementary Fig. S6D). As to be expected, completing the degenerate motif in the p63 linker region to a canonical class II SH3 domain binding motif increased the affinity to the iASPP CTD with a K_D_ of 1.7 µM (Supplementary Fig. S5E). Mutation of the key interacting residues R337 and R340 drastically reduced the binding of ΔNp63α to the iASPP CTD (Fig. 6E; Supplementary Fig. S5F), emphasizing their importance for binding.

To verify that the interaction of the p63 peptide with the SH3 domain within the CTD of iASPP also occurs in solution and is not an artifact of the fusion and the crystal lattice, we titrated the peptide to a ^15^N-lableled iASPP CTD domain and monitored the interaction by NMR spectroscopy (Supplementary Fig. S6A). The same approach was successfully applied for the p53 linker region before [6]. The observed chemical shift perturbations (CSPs) were in the fast to intermediate exchange regime (Supplementary Fig. S6B). The spectra could not be completely assigned due to a strong peak overlap of the ankyrin repeats, but the SH3 domain was mostly covered. The strongest CSPs were found in the SH3 motif binding site suggesting that the p63 linker peptide indeed interacts with the iASPP SH3 domain (Supplementary Fig. S6C, D) also in solution. CSPs of selected residues in the SH3 domain were used to determine the affinity. The individual dissociation constants were in the range between 64 μM to 105 μM, confirming the previous ITC measurements (Supplementary Fig. S6E, F).

### The expression ratios of full-length p63 and the ΔExon8 variant differ between tissues

**Fig. 7:**
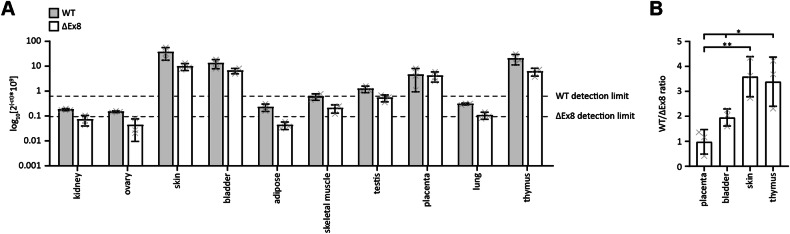
Comparison of expression levels of p63 WT and the ΔExon8 (ΔEx8) variant. **A** The mRNA levels corresponding to WT and ΔExon8mRNA were determined by quantitative RT-PCR in the indicated tissues. Both variants were only detectable in RNA preparations from skin, bladder, placenta and thymus. The detection limit was determined using a water sample for each primer pair. **B** The ratios of WT and ΔExon8 levels in the four tissues containing detectable p63 mRNA were significantly different between placenta on the one hand vs. bladder, skin and thymus on the other hand. Whereas both isoforms were almost equally expressed in placenta, WT was predominant in the other tissues.

The experiments so far have shown that the residues in the short ΔExon8 linker region modulate the interaction of p63 with the DNA and with iASPP. This prompted us to investigate whether differences in tissue distribution of the ΔExon8 splice variant and the non-spliced version exist. To address this question, we identified the level of both forms in ten different human tissues by real-time qPCR (Fig. 7). In all tissues tested, the non-spliced variant was more strongly expressed than the ΔExon8 splice form; however, the ratio of both forms varied between tissues. In the placenta, both are expressed almost equally, whereas in skin and thymus, the non-spliced form is approximately three times more abundant. These findings strongly suggest that the two isoforms can be expressed at different levels, depending on tissue-specific regulation.

## Discussion

The obvious role of the linker region is to connect the DBD and OD with a certain spacing that allows for optimal DNA binding as a tetramer. Additionally, it harbours the NLS just N‐terminally of the OD. No other functions were reported for the linker so far. Here, we characterized the importance of the ΔExon8 linker region with respect to its interaction with iASPP and with DNA. The linker region enhances the affinity of p63 to DNA two- to threefold relative to the ΔExon8 version. Our investigation of the distribution of both p63 forms in different tissues has shown that both seem to always co-exist. As both ΔNp63α and ΔNp63α ΔExon8 contain a complete OD, they can form mixed hetero-tetramers. Potentially, the formation of these mixed tetramers can fine-tune the affinity of p63 to DNA which might particularly affect the interaction with weak binding sites. As p63 also forms (preferentially) mixed tetramers with p73 [24–26], these splice forms increase the complexity of transcriptional complexes of p63 even further. The main function of p63 in epithelial tissues is that of a bookmarking factor, preferentially binding not to promoter regions but to enhancer elements [27, 28]. ChIPseq experiments have identified several thousand binding sites for p63 in different cell types [29, 30] and the occupancy of these sites will also depend on the affinity for the DNA sequence. It is tempting to speculate that the differential expression of the two isoforms in different tissues results in differential binding to these sites.

In addition to increasing DNA binding affinity, the linker sequence is also responsible for the interaction with iASPP. As the affinity for iASPP is much weaker for ΔExon8 than for p63 WT, the formation of mixed tetramers also modulates the interaction with iASPP. iASPP is mainly known for its function as inhibitor of p53-induced apoptosis [3] and its deletion induces p53-mediated cell death in cancer cells [31, 32]. However, iASPP is also involved in developmental processes in epithelial tissues where p63 plays a major role [12, 13]. Germline mutations in iASPP cause cardiocutaneous disorder with abnormal hair growth in mice and cattle as well as skin abnormalities in humans [33–36]. Deletion of iASPP in keratinocytes of mice results in squamous epithelium abnormalities and causes open eyelids at birth and delayed wound healing [34]. In human basal epithelial cells, nuclear iASPP co-localises with p63 [33, 37, 38] and it is suggested that iASPP inhibits keratinocyte differentiation [37]. Through binding to the ΔExon8 specific sequence, iASPP could potentially modulate the DNA binding affinity of the non-spliced version, regulating the binding to certain sites. Such a modulation of the DNA binding properties is reminiscent of the binding of iASPP to p53 which does not inhibit DNA binding but changes the transcriptional program [8].

Recently, we could show that iASPP regulates the expression of a sub-set of p63 and AP1 target genes, including genes involved in skin differentiation and inflammation [14]. Direct interaction between AP1 components such as JunD could be traced to a PxxP peptide that interacts with the SH3 domain of iASPP. This is the same site to which p63 can bind via the ΔExon8 specific sequence. This finding predicts that iASPP can either bind to JunD or to p63. How these interactions could regulate the expression of p63 and AP1 target genes mechanistically is currently not understood. The situation becomes even more complicated by the tetrameric state of p63 and the dimeric state of AP1 which can also include different Jun and/or Fos family members, of which only some interact with iASPP.

Interestingly, p63 mutations are frequent in cutaneous melanoma with a clear indication that UV irradiation is responsible as they mainly occur at pyrimidine-pyrimidine sites. One hot spot for mutations in cutaneous melanoma exits at residue R340 [39]. This residue is part of the linker sequence and is a critical residue for contacting the CTD of iASPP (Fig. 6C). If the loss of interaction with iASPP and/or the weakening of the DNA binding affinity by this mutation contribute to cutaneous melanoma development remains to be investigated.

## Material and Methods

### Quantitative real-time polymerase chain reaction (qRT-PCR)

The expression levels of the p63 full length and delex8 were determined in ten different human tissues (cDNA ordered from Zyagen) by real-time qPCR using a thermocycler (CFX Opus 96 Bio-Rad Laboratories). The reaction mixture contained 1 µl each forward and reverse Primer (10 µM) (listed below), 8 µl ddH_2_O, 1 µl cDNA (Cat: #: HD-010-HR random primer, Zyagen) and 14 µl qPCR mixture (final concentrations per well: 75 mM Tris-HCl pH 8.8 (Roth), 20 mM (NH_4_)_2_SO_4_ (Roth), 0.01% Tween-20 (AppliChem), 3 mM MgCl_2_ (Sigma-Aldrich), 0.25% Triton X-100 (AppliChem), 0.2 mM dNTPs (Primetech), 20 U/ml Taq-polymerase (Primetech), 300 mM Trehalose (Roth) and SYBR Green (1:40 000) (Invitrogen). For PCR, the samples were preheated for 2 min at 95 °C, followed by 45 cycles at 95 °C for 20 s, 60 °C for 1 min and 72 °C and 80 °C for each 5 s.

Each sample was analysed in duplicates with additional no-template controls.

Primer sequences for RNA quantification

**Table:** 

Primer Target	Forward Primer [10 µM]	Reverse Primer [10 µM]
p63 full length	CAGGAAGACAGAGTGTGCTG	ACGAAACGGGCGCTTCGTAC
p63 delex8	CAGGAAGACAGAGTGTGCTG	TTCTGACGAAACGCATCACC
36B4	GATTGGCTACCCAACTGTTG	CAGGGGCAGCAGCCACAAA

### Protein expression and purification

Protein expression plasmids were transformed into *E. coli* BL21(DE3) Rosetta cells and incubated on a LB agar plate, supplemented with appropriate selection antibiotics. The following day, cells were transferred into 2xYT medium (supplemented with 100 µM zinc acetate for expression of p53 family domains) and grown at 37 °C until an optical density of ~0.8 was reached. Expression was induced with 1 M IPTG for 16–18 h at either 16 for ASPP CTDs or 18–22 °C for p53 family proteins. Labelled expression of the iASPP CTD for NMR measurements was performed similarly, but in M9 minimal medium instead of 2xYT as described previously [24]. Cells were harvested by centrifugation and resuspended in IMAC A buffer (25 mM Tris pH 7.8, 200 mM NaCl, 20 mM β-mercaptoethanol (β-ME), 5% glycerol, 25 mM imidazole) supplemented with lysozyme (Sigma-Aldrich), RNAse (SigmaAldrich), DNAse (Sigma-Aldrich) and protease inhibitors (Carl Roth). After lysis by sonification and centrifugation at 4 °C, proteins were subjected to immobilized metal affinity chromatography (IMAC) purification using HiTrap IMAC Sepharose FF columns (Cytiva). After washing with IMAC A buffer, bound proteins were eluted with IMAC B buffer (IMAC A buffer with 300 mM imidazole). GST-tagged proteins were then directly loaded onto a GSTrap FF column (Cytiva), washed with GAC A buffer (25 mM TRIS pH 7.8, 400 mM NaCl, 20 mM β‐ME) and eluted with GAC B buffer (GAC A buffer with 10 mM reduced L‐Glutathione). All other proteins were treated with TEV protease (1:50 w/w) for cleavage of the purification tag and simultaneously dialysed against IMAC A buffer (supplemented with 50 mM imidazole for p53 family DBD-TD constructs) overnight. Cleaved tags and TEV protease were removed by reverse IMAC. All proteins were further purified by ion exchange chromatography on a HiTrap Q HP cation exchange column (for ASPP CTDs) or a HiTrap Heparin HP column (for p53 family DBD-TDs). If necessary, protein solutions were diluted with IEX A buffer (25 mM HEPES pH 7.0, 50 mM NaCl, 5% Glycerol, 20 mM β‐ME; supplemented with 10 μM zinc acetate for p53 family DBD-TDs) to reduce the salt concentration below 100 mM prior loading. Bound protein was eluted with a salt gradient from 50 mM to 1 M NaCl mixing IEX A buffer with IEX B buffer (IEX A buffer with 1 M NaCl). For the final purification step, proteins were loaded onto a Superdex 75 16/600 or a Superdex 200 16/600 column (Cytiva), equilibrated with SEC buffer (25 mM HEPES pH 7.5, 150 mM NaCl, 0.5 mM TCEP). Monodisperse peak fractions were pooled, concentrated by centrifugation and snap frozen in liquid nitrogen for storage at −80 °C until usage. All purification steps were performed at 4 °C and with the help of an ÄKTA purifier chromatography system.

### Pulldowns

Purified GST-tagged ASPP CTDs or GST alone were immobilized on magnetic glutathione agarose beads for 1 h at 4 °C in pulldown buffer (50 mM Tris pH 8.0; 150 mM NaCl; 0.1% Tween-20). For DNA pulldowns, biotinylated double-stranded DNA was immobilized on streptavidin agarose beads (GE Healthcare). Concurrently, p63 variants were in-vitro translated using the TNT T7 Quick Coupled Transcription/Translation System (Promega) at 30 °C for 90 min. After centrifugation for 15 min at 13,000 xg and preparation of input samples, the protein was incubated with the loaded beads at 4 °C for 3 h. Following four washing cycles with pulldown buffer, bound p63 was eluted with boiling sample buffer (1x NuPAGE LDS buffer (Thermo Fisher Scientific), supplemented with 50 mM DTT) and analysed by western blot. For quantification of the pulldown efficiency, each pulldown sample was normalized on its corresponding input sample. All pulldowns were performed in triplicates.

### ELISA

Biotinylated dsDNA oligonucleotides in assay buffer (DPBS (Gibco), 0.1% Tween-20, 10% Superblock (Thermo Fisher Scientific)) were immobilized on streptavidin pre-coated 96-well plates (Thermo Fisher Scientific). Purified His-tagged p63 DBD-TD proteins (AA123–416) were added to the well plate and incubated for 1 h. After four washing cycles with wash buffer (assay buffer without Superblock) and incubation with an HRP-coupled anti-His antibody, TMB solution was added to each well before the reaction was stopped with 2 M sulphuric acid after 6 min and the absorption was measured. Measurement were performed as triplicates at RT. Binding affinities and corresponding 95% confidence intervals were determined by fitting the data points with a non‐linear, least squares regression using a single‐exponential one‐site binding model.

### ITC

Isothermal titration calorimetry (ITC) measurements were performed using a VP-ITC device (MicroCal). Purified proteins expressed in *E. coli* and oligopeptides were dialysed against ITC buffer (25 mM HEPES pH 7.5, 150 mM NaCl, 500 µM TCEP) overnight at 4 °C. Prior measurement, proteins were centrifuged and degassed to remove any aggregates and air bubbles from the samples. Resulting binding curves were corrected for the dilution heat of the titrant and analysed using the software NITPIC and SEDPHAT, assuming AB hetero association. Final figures were prepared with GUSSI. All measurement parameters and determined thermodynamic parameters are listed in Table S1.

### Thermal shift assay

Melting temperatures of purified p63 DBD-linker fusions (amino acids 123–351) were determined via thermal shift assay. Samples were thawed and centrifuged at 13,000 xg for 5 min at 4 °C before mixing them with SYPRO Orange (Thermo Fisher Scientific, 1:500 diluted in assay buffer) and diluting them to an end concentration of 20 µM with assay buffer (50 mM BIS‐TRIS pH 6.8, 100 mM NaCl and 0.5 mM TCEP). Using a QuantStudioTM 5 cycler (Bio-rad), the measurement was performed at an initial temperature of 4 °C which was gradually increased to 99 °C with a rate of 0.1 °C/s. The fluorescence signal was detected and the melting temperature was determined by calculating the negative first deviation (-dF/dT) with GraphPad Prism 8.

### Analytical size exclusion chromatography

p63 isoforms with WT or ΔExon8 linker region were in-vitro translated at 30 °C for 90 min using the TNT T7 Quick Coupled Transcription/Translation System (Promega). To analyse their oligomerization status, proteins were injected onto a Superose 6 3.2/300 column, equilibrated with SEC running buffer (50 mM Tris pH 7.5; 150 mM NaCl; 1 mM DTT) and connected to an ÄKTA purifier chromatography system at 4 °C. Collected fractions were analysed by WB using an α-Myc antibody.

### Cell culture

The human non-small cell lung carcinoma cell line H1299 was cultured in RPMI 1640 medium (Thermo Fisher Scientific) supplemented with 10% Fetal Bovine Serum (Capricorn Scientific), 100 U/ml penicillin and 100 µg/ml streptomycin (Thermo Fisher Scientific) and incubated in a humidified 5% CO_2_ atmosphere at 37 °C. Cells were regularly passaged and tested for mycoplasma contamination. Transfections with Lipofectamine 2000 (Thermo Fisher Scientific) transfection reagent followed the manufacturers’ protocols.

### Luciferase reporter assay

H1299 cells were transiently transfected with pRL-CMV (Promega), pGL3 Basic with a PUMA or K14 promotor and an empty vector control or the indicated Myc-tagged p63 variants on a pcDNA3.1(+) plasmid. Cells were harvested 24 h after transfection and resuspended in fresh medium, before taking input samples and transferring the remaining lysate into a 96-well plate. The luciferase reporter assay was performed using the Dual-Glo luciferase assay system (Promega) following the manufacturer’s instructions. All measurements were performed in technical quadruplicates and biological triplicates. After calculating the Firefly to Renilla luciferase activity ratio for each technical replicate and determining the mean, each biological replicate was normalized to the empty vector control.

For the displacement assay, H1299 cells were transiently transfected with pRL-CMV (Promega), pBV-Luc BDS-2 3x WT (pBDS-2) and either an empty vector control, Myc-p53 alone or with increasing amount of indicated Myc-tagged p63 variants. The total amount of transfected plasmid DNA was kept constant by adding an appropriate amount of empty vector, if necessary. Cell harvesting and assay execution followed the protocol described above.

### SDS Page and Western blotting

Samples subjected to SDS PAGE were boiled for 5 min at 95 °C before being loaded onto a 4–15% Mini-PROTEAN TGX gel (Bio-rad). Precision Plus Protein Dual Color Standards marker (Bio-rad) was used as a size reference. After separation, proteins were transferred onto a PVDF membrane using the TransBlot Turbo transfer system (Bio-rad) according the manufacturers’ protocol. Following incubation with the respective primary and HRP-coupled secondary antibodies, blots were imaged using Amersham ECL Prime western blotting detection reagent and the ChemiDoc imaging system (Bio-Rad). Signal intensity was quantified using the Image Lab 6.1 software (Bio-rad). For pulldown assays, blots were additionally stained with Ponceau staining to visualize the protein level of the immobilized bait protein.

Antibodies used for Western blotting

**Table:** 

Primary antibodies	Clone number	Manufacturer
anti-Myc	4A6	Sigma-Aldrich
anti-vinculin	7F9	Santa Cruz
Secondary antibody	Clone number	Manufacturer
Anti‐Mouse IgG (Fab specific)–Peroxidase antibody produced in goat	A9917	Sigma‐Aldrich

### Statistical analysis

Unless otherwise specified, experiments were performed in triplicates to determine the mean and standard deviation. Comparison of independent data sets was performed using one-way analysis of variance (ANOVA) followed by Tukey’s HSD post hoc tests. P values denoting statistical significance are represented as *P ≤ 0.05; **P ≤ 0.01; ***P ≤ 0.001. All statistical analyses were performed using GraphPad Prism 8.

### Cloning

For transient expression in mammalian cells and in-vitro translation of N-terminally Myc-tagged p53, p63 and p73 isoforms, PCR-generated inserts were introduced in pcDNA3.Myc, a derivative of pcDNA3.1(+) (Thermo Fisher Scientific) with a Myc-tag between HindIII and BamHI sites [19]. The inserts lack the intrinsic start codon to avoid expression of untagged proteins via alternative translation initiation skipping the Myc-tag.

For recombinant expression of the ASPP CTDs and p63-iASPP CTD fusion in E. coli, PCR-generated inserts were introduced in pET-15b-His10-TEV (N-terminal His10-tag followed by TEV protease cleavage site [19]) by subcloning using BamHI and XhoI restriction sites.

For expression of the p53 family DBDs, PCR-generated inserts were introduced in pET-15b (Novagen) by subcloning using NcoI/BamHI and XhoI restriction sites, simultaneously adding either a N-terminal His10-tag followed by a TEV protease cleavage site (HHHHHHHHHHDYDIPTTENLYFQGS) or a C-terminal TEV protease cleavage side followed by a His6-tag (AENLYFQGHHHHHH), as described before [19].

Any mutations were subsequently introduced by site-directed mutagenesis.

### NMR

For the p63 DBD-linker fusions, 100 µM purified [^15^N]-labelled proteins in NMR buffer (50 mM BIS‐TRIS pH 6.8, 100 mM NaCl and 0.5 mM TCEP) were supplemented with 150 µM DSS and 5% D_2_O before recording two separate [^1^H-^15^N]-BEST-TROSY HSQC spectra for superposition.

For analysing the interaction between the iASPP CTD and p63 peptide, purified labelled proteins and unlabelled peptides were buffer exchanged into the NMR buffer (25 mM HEPES pH 7.0, 50 mM NaCl and 5 mM DTT) by dialysis. The samples were prepared for measurement by supplementing with 150 µM DSS and 5% D_2_O as well as 1x protease inhibitor. A sample with 1 mM [^15^N,^13^C]-iASPP CTD was prepared to record [^1^H-^15^N]-BEST-TROSY HSQC, HNCACB, HN(CA)CO and HN(CO)CACB spectra for backbone assignment. For the NMR titration experiment, one NMR sample with 100 µM [^15^N]-iASPP CTD alone and another with [^15^N]-iASPP CTD and 500 µM p63 linker peptide was prepared. [^1^H-^15^N]-BEST-TROSY HSQC spectra of both samples were recorded at 288 K as the endpoints of the titration. Then the next samples were prepared by mixing the previous ones. This process was repeated until sufficient titration points were recorded. All spectra were assigned with Sparky 3.114. HNCACB, HN(CA)CO and HN(CO)CACB spectra were used for the backbone assignment. The [^1^H-^15^N]-BEST-TROSY HSQC spectra of the titration experiment were assigned on the basis of this backbone assignment. Chemical shift perturbations (CSPs) were extracted and plotted for selected residues against the concentration of the p63 peptide. The individual equilibrium dissociation constants were determined with a non-linear, least squares regression using a single-exponential one-site binding model (GraphPad Prism 8).

All NMR samples were measured at 288 K using Bruker Avance spectrometers (proton frequencies of 600 MHz, 800 MHz and 950 MHz).

### Crystallization

Freshly purified p63-iASPP CTD fusion protein was concentrated in crystallization buffer (25 mM HEPES pH 7.5, 150 mM NaCl and 0.5 mM TCEP) to 8–10 mg/ml. Crystallization was performed with the sitting and hanging drop vapor diffusion method at 20 °C. The final optimized crystallization condition was 2 M sodium formate and 0.1 M TRIS pH 7.5. Before flash cooling in liquid nitrogen, crystals were cryo-protected with mother liquor supplemented with 25% ethylene glycol. Diffraction data were collected at Swiss Light Source, processed with XDS [40] and scaled with Scala of the CCP4 suite [41]. The structures were initially solved by molecular replacement using Phaser [42] and the deposited coordinates of the iASPP CTD (PDB code: 2VGE) was used for manual model rebuilding (COOT) [43] and REFMAC for structure refinement [44]. The final structures were verified for correctness of the geometry by Molprobity [45]. Data collection and refinement statistics are shown in Table S2.

## Supplementary information

Supplementary Figure S1

Supplementary Figures and Figure Legends

Table S1

Table S2

## Data Availability

All data are fully available upon request. The PDB accession code for the crystal structure is 9GFO.
