# Supplementary material for: Alternative splicing in the DBD linker region of p63 modulates binding to DNA and iASPP in vitro
Source: Cell Death Dis. 2025 Jan 6;16(1):4. doi: 10.1038/s41419-024-07320-2 (PMC11704248; doi:10.1038/s41419-024-07320-2)

**uncropped western blots for Fig. 2B**

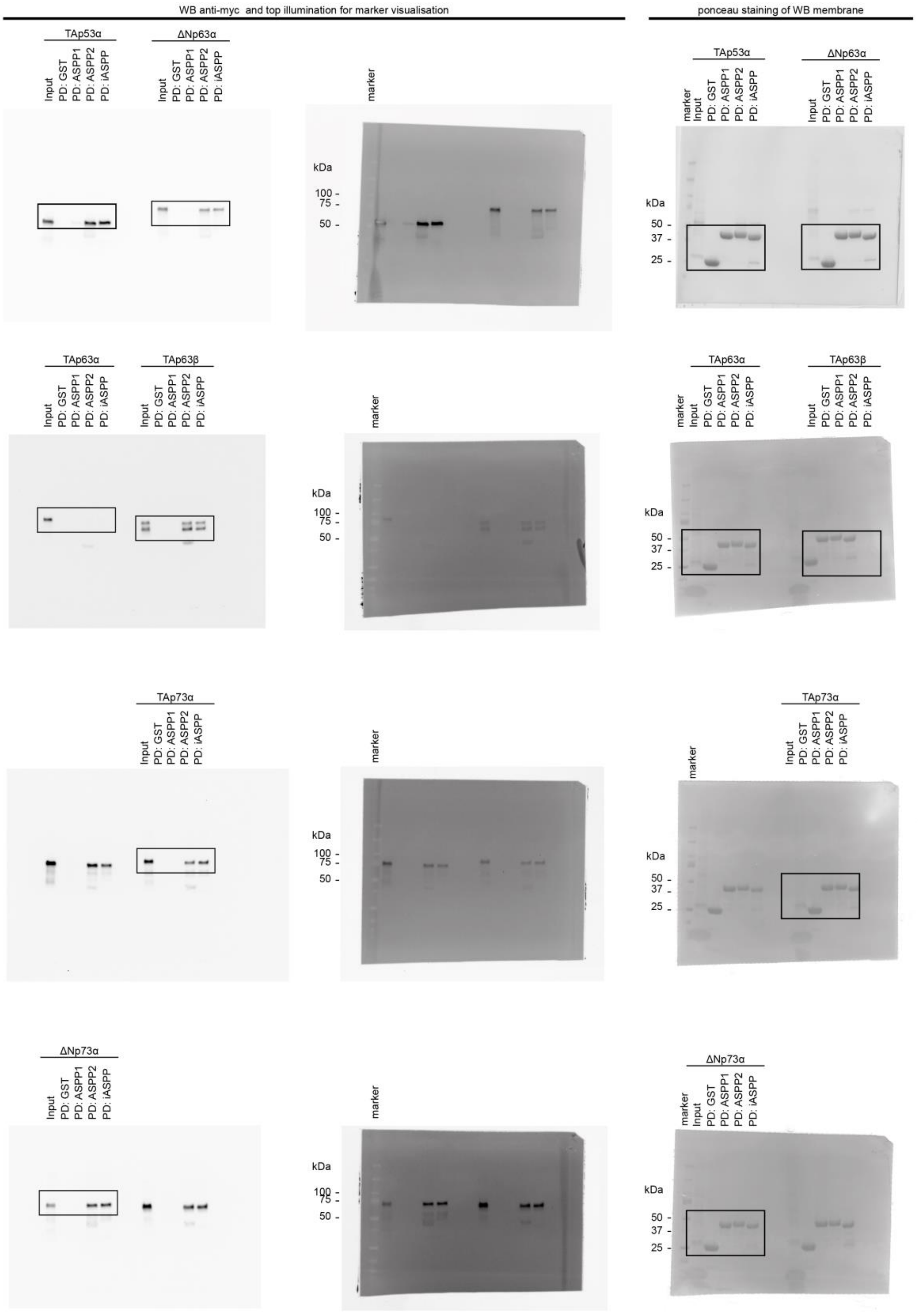

## uncropped western blots for Supplementary Fig. S2A

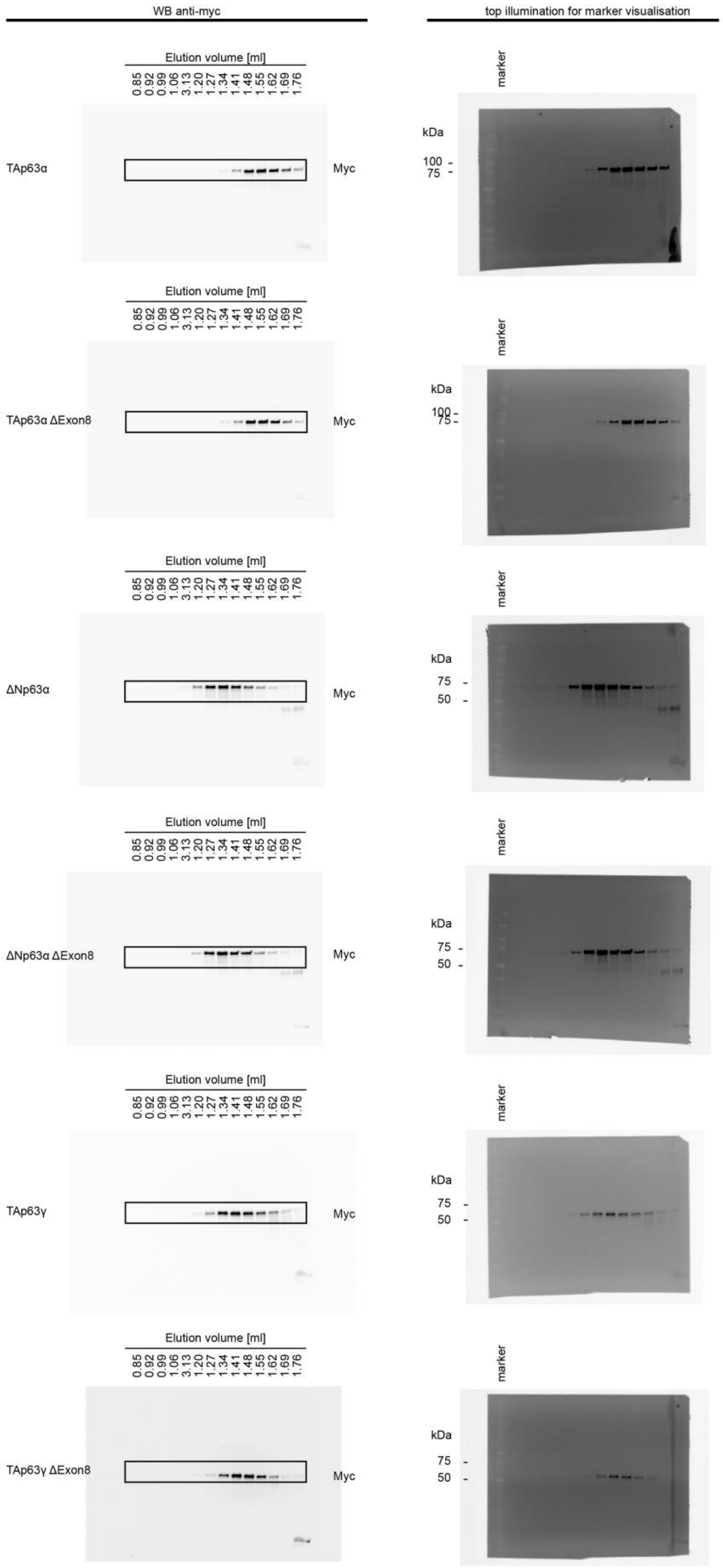

**uncropped western blots for Supplementary Fig. S2D**

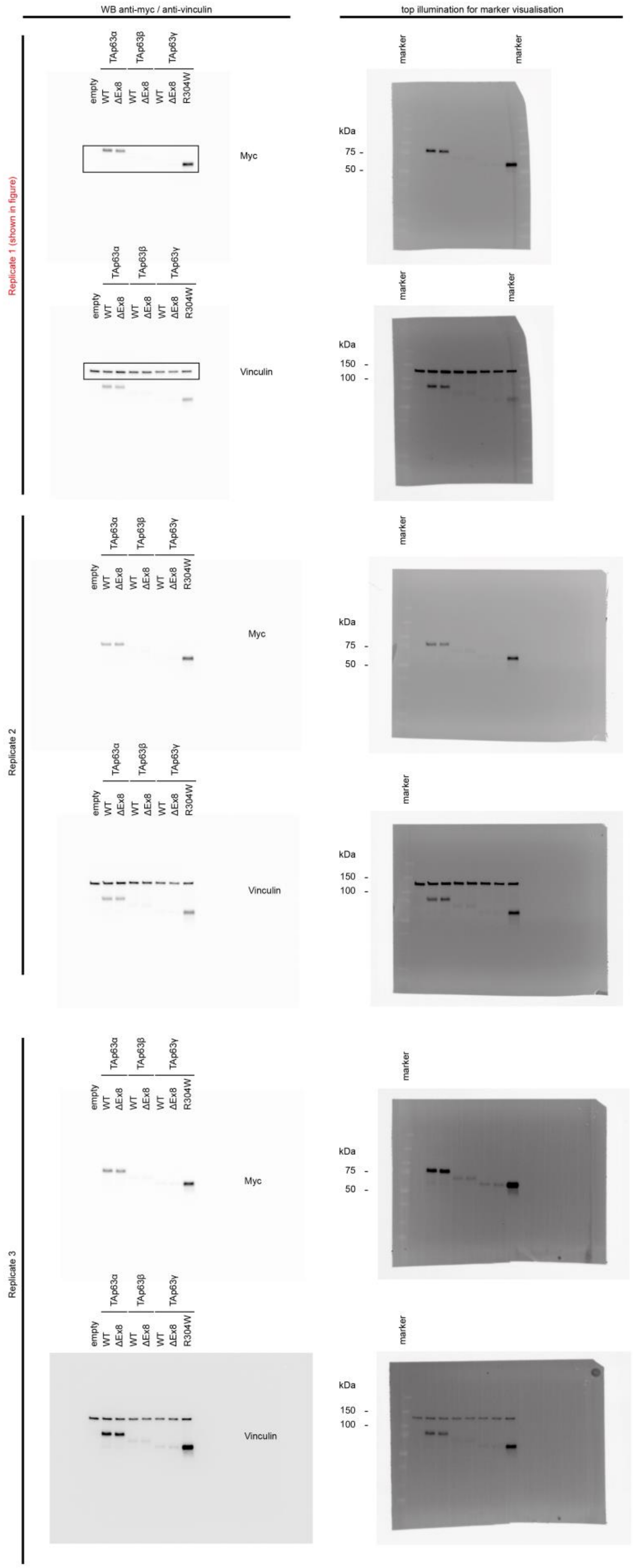

**uncropped western blots for Supplementary Fig. S2E**

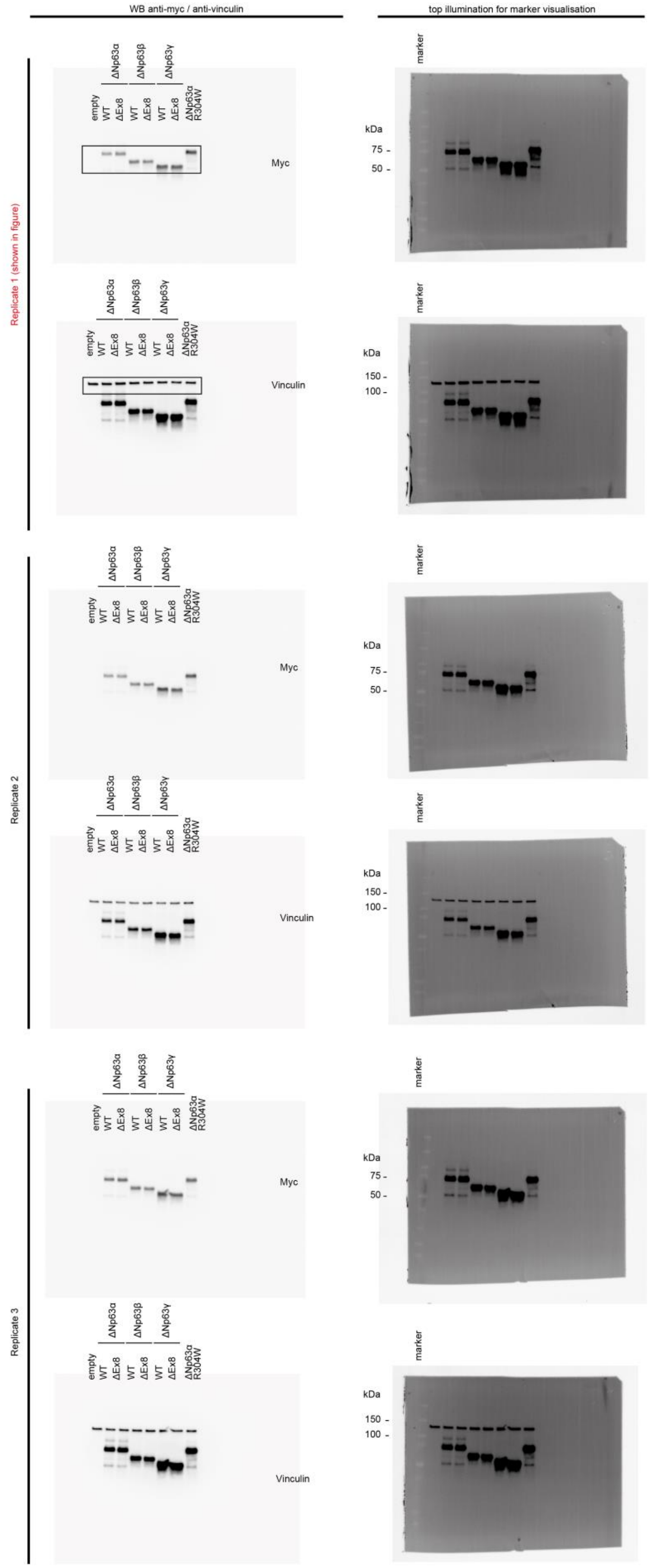

**uncropped western blots for Supplementary Fig. S2F**

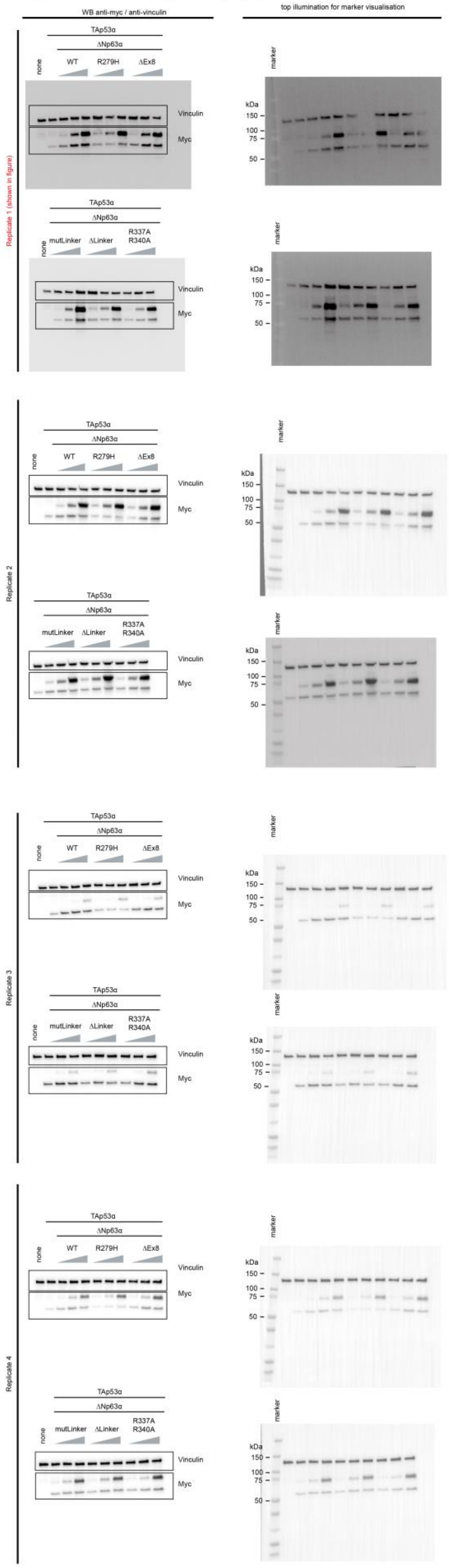

**uncropped western blots for Supplementary Fig. S2G**

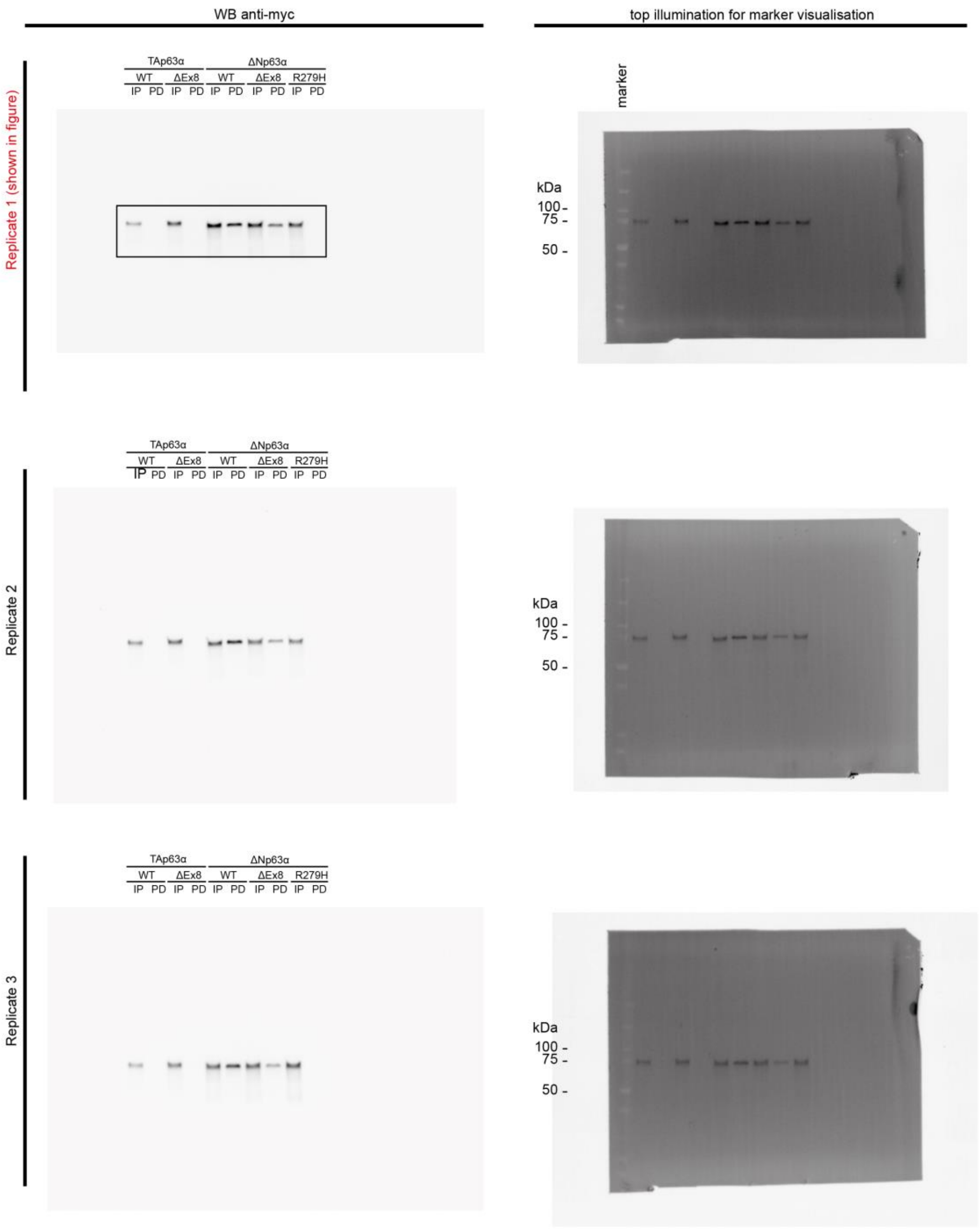

**uncropped western blots for Supplementary Fig. S3A**

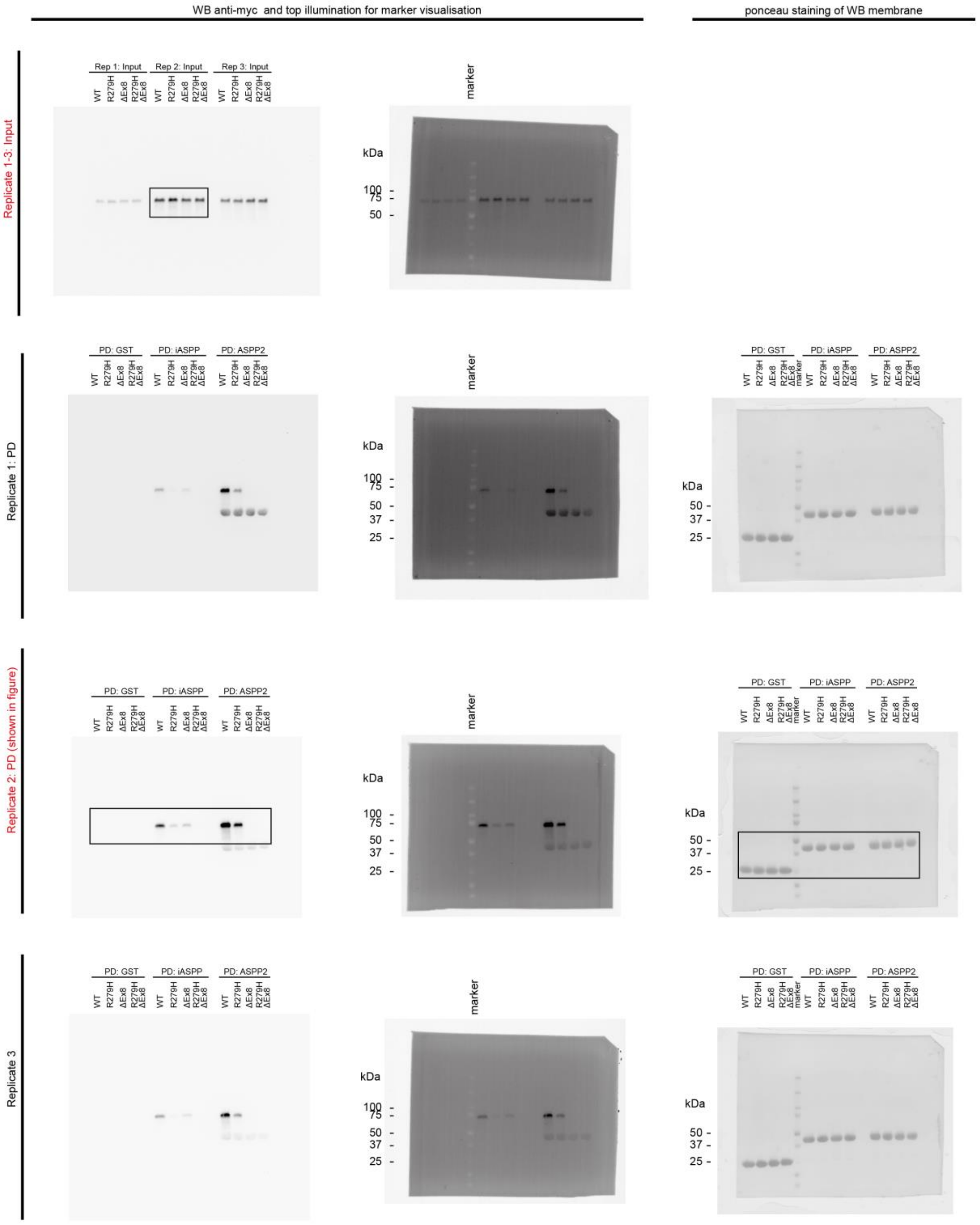

**uncropped western blots for Supplementary Fig. S3B**

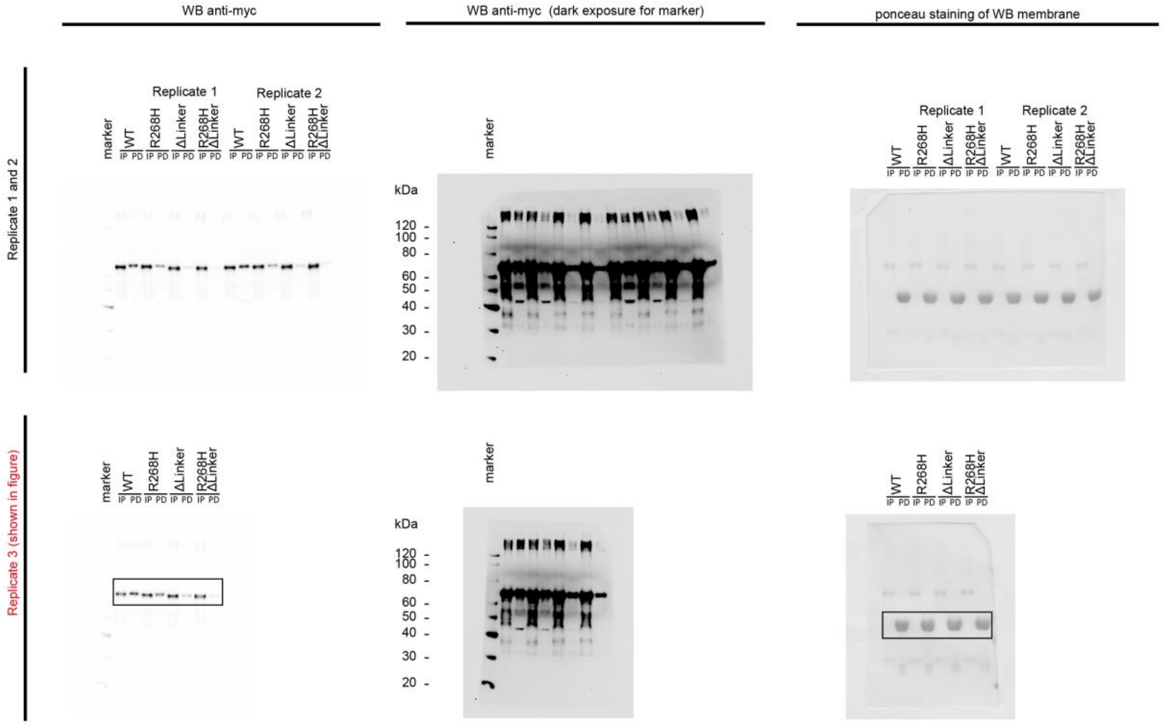

# uncropped western blots for Supplementary Fig. S5F

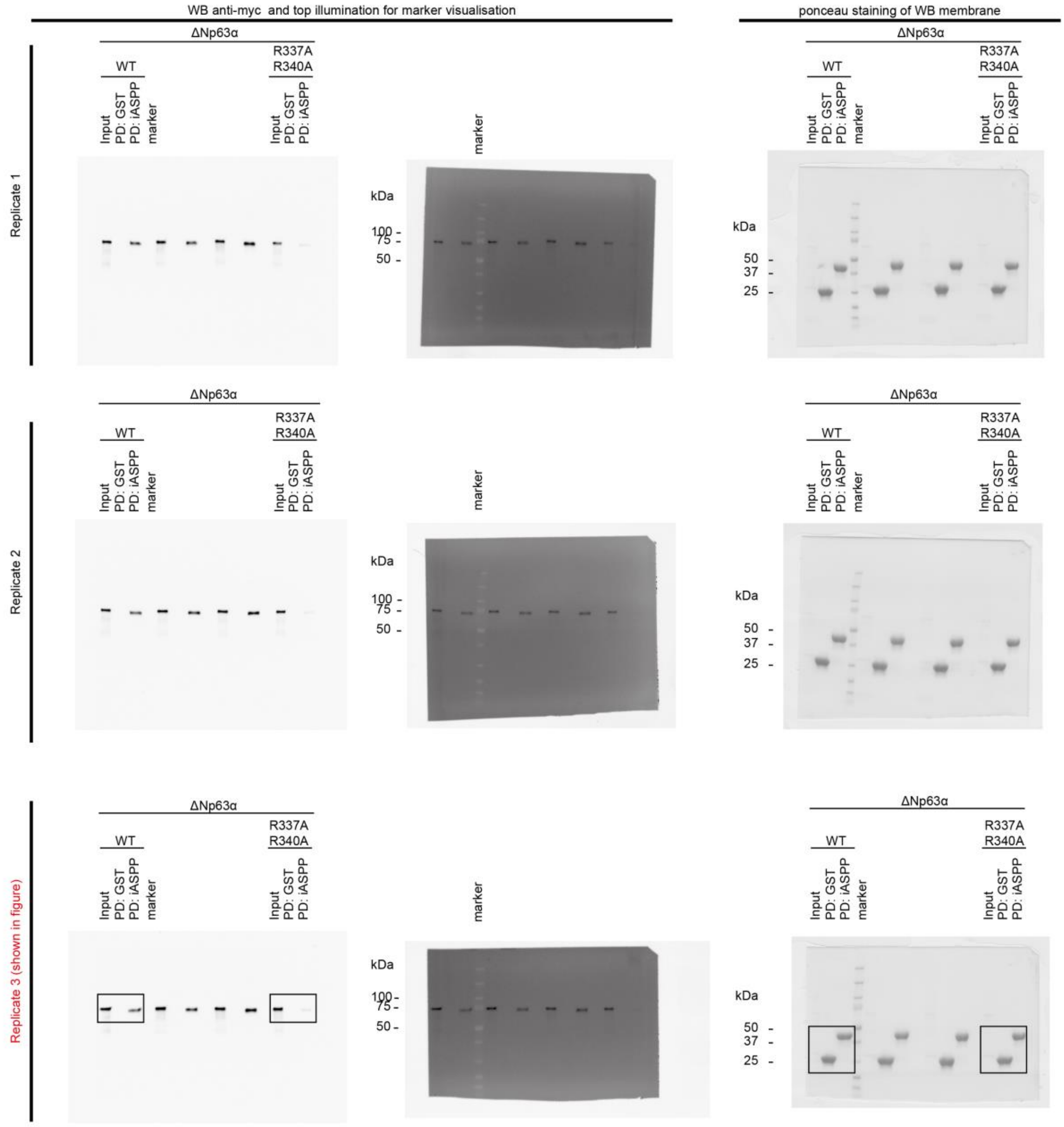

Supplement: Supplementary file 1 — Supplementary Figure S1 [file 41419_2024_7320_MOESM1_ESM.pdf]
