# Supplementary material for: Alternative splicing in the DBD linker region of p63 modulates binding to DNA and iASPP in vitro
Source: Cell Death Dis. 2025 Jan 6;16(1):4. doi: 10.1038/s41419-024-07320-2 (PMC11704248; doi:10.1038/s41419-024-07320-2)
Supplement: Supplementary file 2 — Supplementary Figures and Figure Legends [file 41419_2024_7320_MOESM2_ESM.pdf]

Supplementary Figures and Figure legends

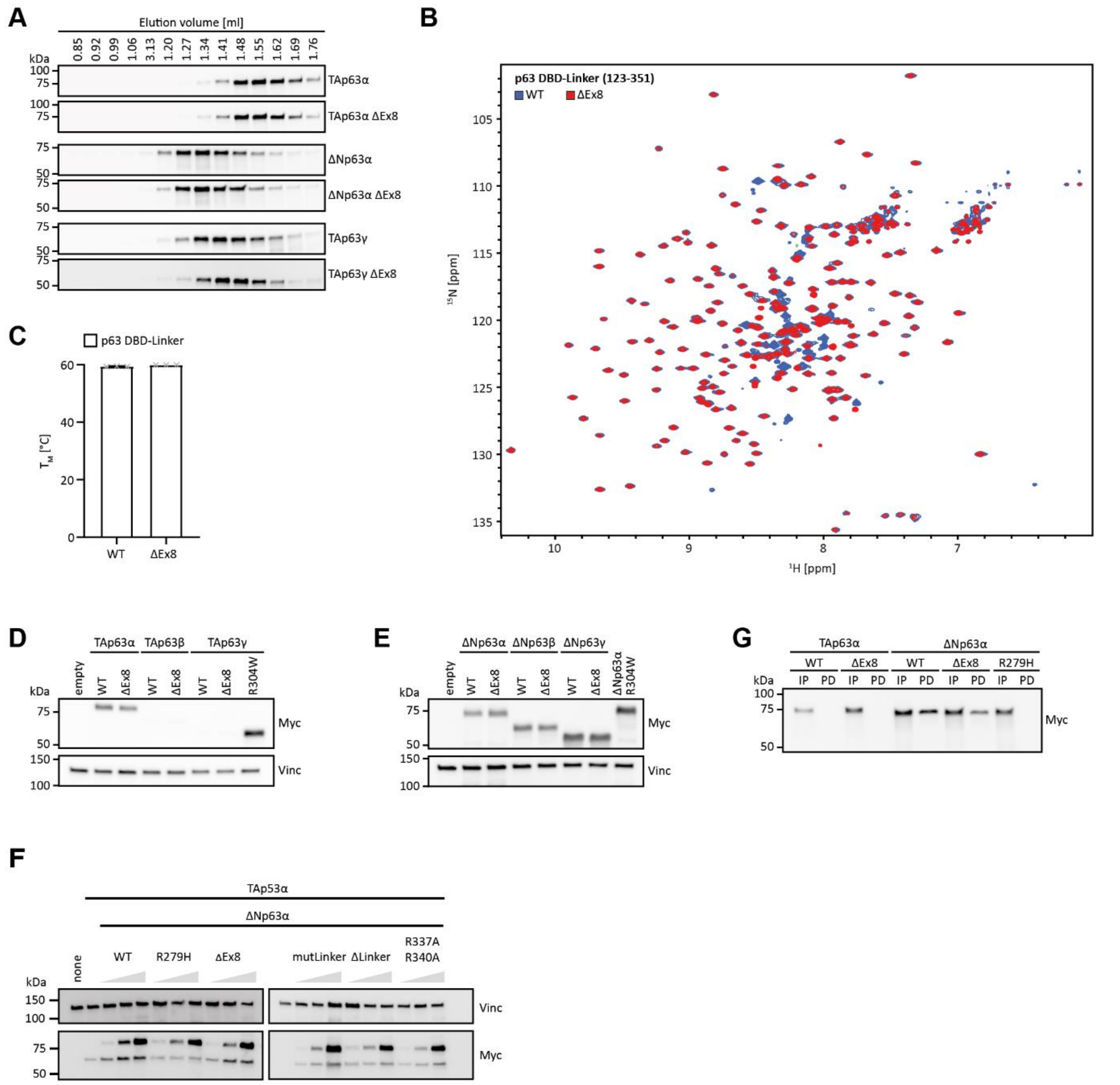

SupMat Figure S2 (A) SEC of p63 isoforms with either the WT or ΔExon8 (ΔEx8) linker region. N-terminally Myc-tagged proteins were *in-vitro* translated using RRL and lysates were loaded onto a Superose 6 SEC column. Collected fractions were analysed for p63 by WB using an α-Myc antibody. An elution volume of 0.850 ml corresponds to the void volume of the column (2.4 ml bed volume). **(B)** Overlay of [^{1}H-^{15}N]-BEST-TROSY HSQC NMR spectra of [^{15}N]-labelled p63 DBD-linker fusions (amino acids 123-351) with either the WT (blue) or the ΔEx8 (red) linker sequence **(C)** Melting temperature of purified p63 DBD-linker fusions (amino acids 123-351) with either the WT or the ΔEx8 linker sequence (Mean ± SD. n=3). **(D)** Expression levels of the transiently transfected proteins in the luciferase reporter assay from Figure 3B were determined by WB using an α-Myc antibody. Vinculin served as loading control. **(E)** Expression levels of the transiently transfected proteins in the luciferase reporter assay from Figure 3C were determined by WB using an α-Myc antibody. Vinculin served as loading

control. **(F)** Expression levels of the transiently transfected proteins in the luciferase reporter assay from Figure 3D were determined by WB using an α-Myc antibody. Vinculin served as a loading control. **(G)** Representative WB of the DNA pull-down assay from Figure 3E.

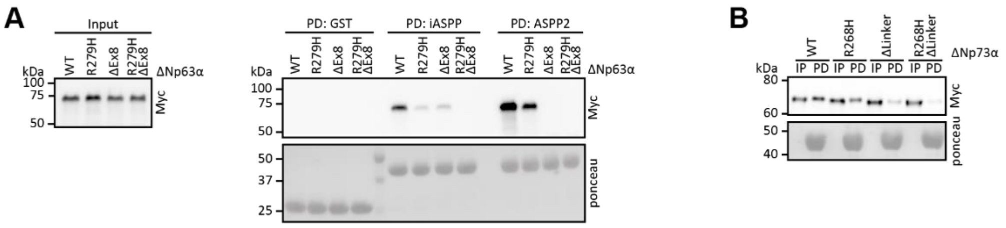

SupMat Figure S3 (A) Representative WB of the DNA pull-down assay from Figure 4A. The bait protein in the PD samples was detected by ponceau staining of the WB membrane. **(B)** Representative WB of the DNA pull-down assay from Figure 4B. The bait protein in the PD samples was detected by ponceau staining of the WB membrane.

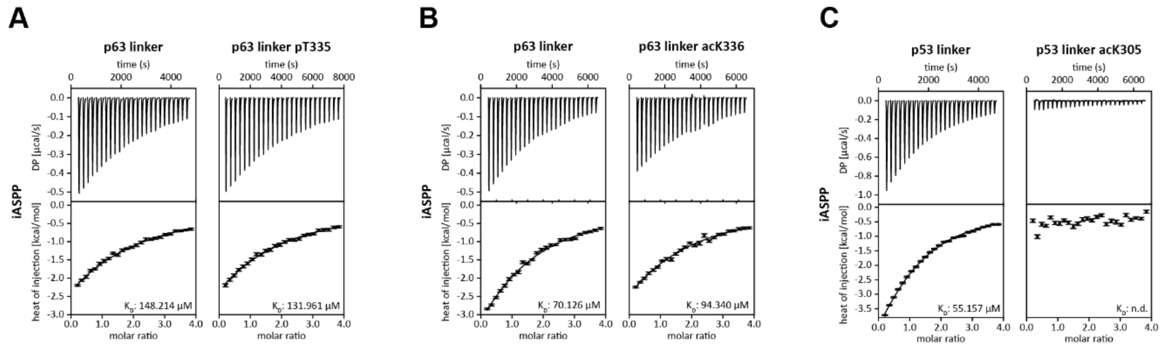

SupMat Figure S4 (A) ITC results of the p63 linker WT and pT335 peptides titrated to iASPP CTD. **(B)** ITC results of the p63 linker WT and acK336 peptides titrated to iASPP CTD. **(C)** ITC results of the p53 linker WT and acK305 peptides titrated to iASPP CTD. For all titration experiments the raw titration profiles are displayed in the top diagrams and integrated heat in the bottom diagram. Best fit of single-site binding model is shown as a solid black line with the resulting K_D.

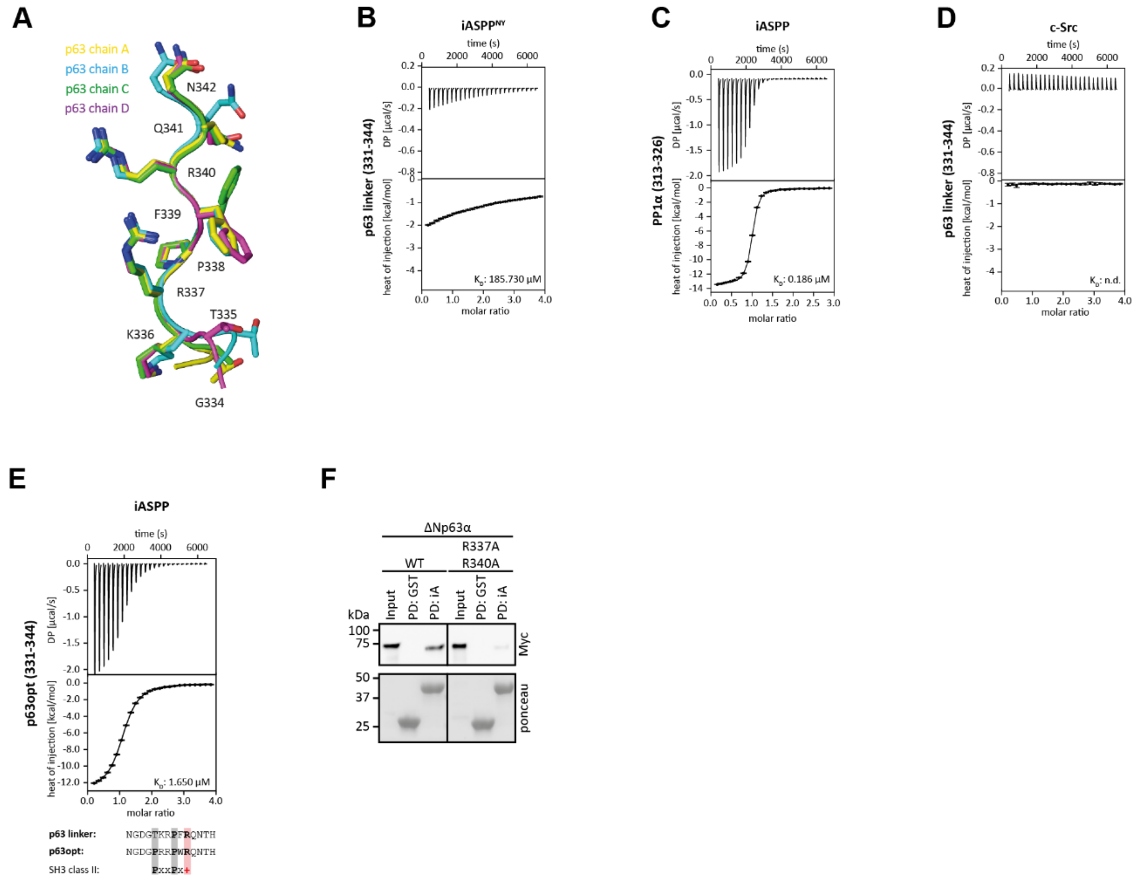

SupMat Figure S5 (A) Superimposition of the four p63 peptide chains (A to D) found in the asymmetric unit of the solved crystal structure. **(B)** ITC results of the p63 linker peptide titrated to the N813A Y814A iASPP double mutant. **(C)** ITC based interaction study of a peptide derived from PP1α (amino acids 313-326) with the iASPP CTD. **(D)** ITC results of a titration of the p63 linker peptide to the Src kinase SH3 domain. **(E)** ITC results of an optimized p63 linker peptide (p63opt) titrated to iASPP CTD. Alignment of the p63 linker and p63opt peptide with the SH3 class II motif is shown below the titration. For all ITC experiments the raw titration profiles are displayed in the top diagrams and integrated heat in the bottom diagram. Best fit of single-site binding model is shown as a solid black line with the resulting K_D. **(F)** Representative WB of the pull-down assay from Figure 6E. The bait protein in the PD samples was detected by ponceau staining of the WB membrane.

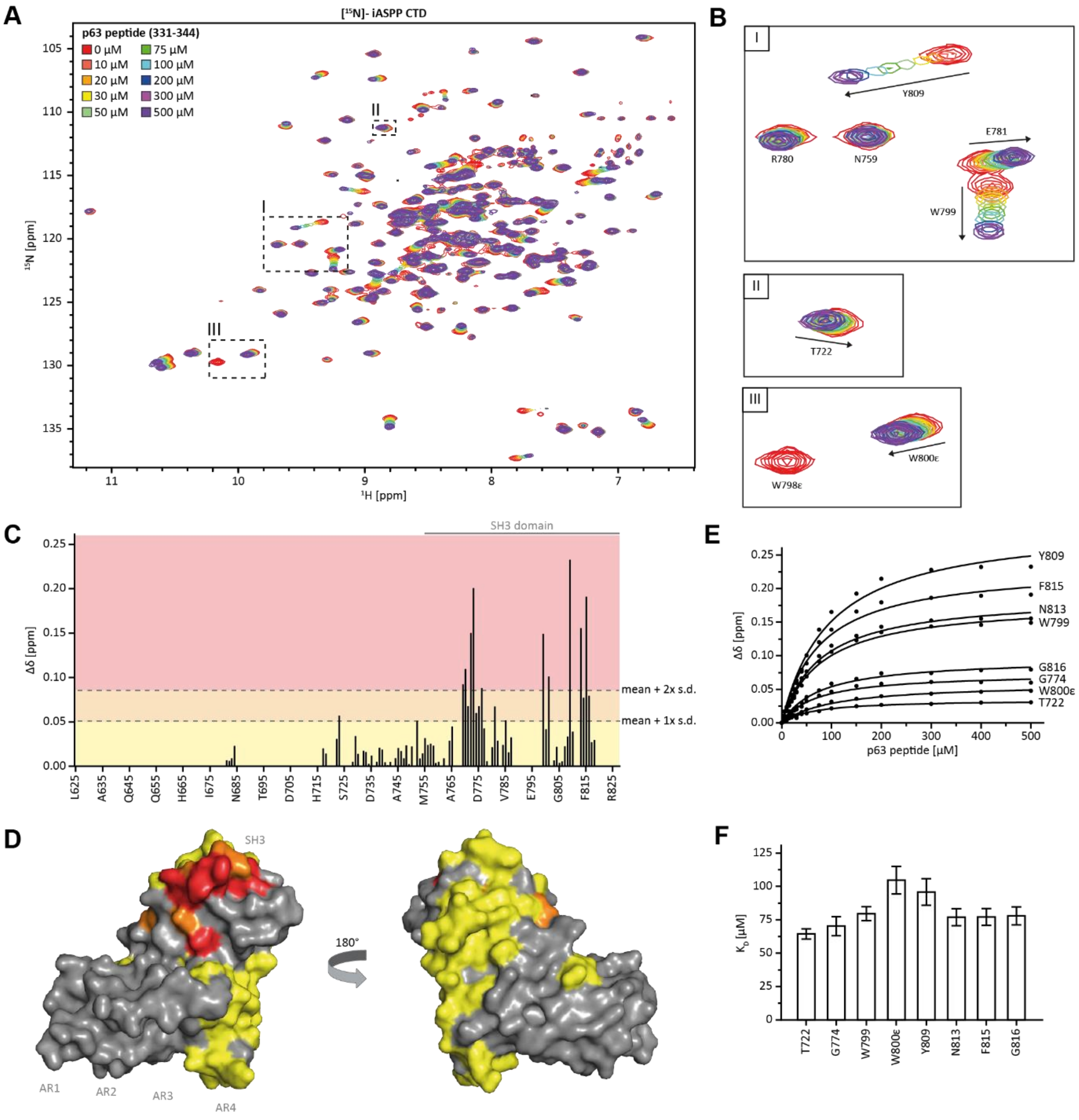

SupMat Figure S6 (A) Overlay of [^{1}H-^{15}N]-BEST-TROSY HSQC NMR spectra of [^{15}N]-labelled iASPP CTD titrated with increasing amounts of unlabelled p63 linker peptide (red to purple). **(B)** Three sections (I, II and III) of the overlay in (A) with peaks corresponding to residues of the SH3 domain interaction surface are shown in detail. Chemical shifts of indicated iASPP SH3 domain residues were in the fast and intermediate regime, highlighted by black arrows. **(C)** Chemical shift perturbations (CSPs, Δδ) of assigned iASPP CTD residues upon addition of 500 µM p63 linker peptide displayed in a bar diagram. CSPs of individual residues were categorized by comparison to the overall mean CSP and SD and colour coded accordingly. The boundaries of the SH3 domain are indicated. **(D)** CSPs of iASPP CTD residues upon addition of 500 µM p63 linker peptide are mapped on its structure according to the colour code in (C). Due to strong peak overlap, the iASPP CTD spectra could not be assigned completely. Unassigned residues are shown in grey (PDB: 2VGE). **(E)** CSPs (Δδ) of several iASPP SH3 domain residues upon titration of p63 peptide were plotted against the p63 peptide concentration and fitted with a one-site binding model for each individual residue to determine the K_D. **(F)** Bar diagram shows the K_D derived from the fitted curves in (E). The error bars correspond to the standard deviation of the fit function.
