# Supplementary material for: Alternative splicing in the DBD linker region of p63 modulates binding to DNA and iASPP in vitro
Source: Cell Death Dis. 2025 Jan 6;16(1):4. doi: 10.1038/s41419-024-07320-2 (PMC11704248; doi:10.1038/s41419-024-07320-2)
Supplement: Supplementary file 3 — Table S1 [file 41419_2024_7320_MOESM3_ESM.docx]

Experimental set-up and determined thermodynamic parameters including the 95% confidence intervals of ITC measurement
