# Supplementary material for: Alternative splicing in the DBD linker region of p63 modulates binding to DNA and iASPP in vitro
Source: Cell Death Dis. 2025 Jan 6;16(1):4. doi: 10.1038/s41419-024-07320-2 (PMC11704248; doi:10.1038/s41419-024-07320-2)
Supplement: Supplementary file 4 — Table S2 [file 41419_2024_7320_MOESM4_ESM.docx]

| **Structure** | **iASPP p63** |
| --- | --- |
| **PDB accession code** | 9GFO |
| **Data Collection** |  |
| Resolution^a^ (Å) | 47.24-2.40 (2.47-2.40) |
| Spacegroup | *P*22_­1_2 |
| Cell dimensions | *a* = 77.1, *b* = 94.1, *c* = 179.3 Å |
|  | *α, β,* *γ* = 90.0° |
| No. unique reflections^a^ | 51,860 (4,431) |
| Completeness^a^ (%) | 99.9 (100.0) |
| I/σI^a^ | 13.4 (1.8) |
| R_merge_^a^ | 0.062 (0.899) |
| CC (1/2) | 0.997 (0.706) |
| Redundancy^a^ | 6.0 (6.2) |
| **Refinement** |  |
| No. atoms in refinement (P/O)^b^ | 6,379/ 127 |
| B factor (P/L/O)^b^ (Å^2^) | 78/ 62 |
| R_fact_ (%) | 21.4 |
| R_free_ (%) | 23.9 |
| rms deviation bond^c^ (Å) | 0.010 |
| rms deviation angle^c^ (°) | 1.2 |

**Data collection and refinement statistics.**

^a^ Values in brackets show the statistics for the highest resolution shells.

^b^ P/O indicate protein and others (water and solvent molecules), respectively.

^c^ rms indicates root-mean-square.
